# Cross-Talk Between Intestinal Microbiota and Host Gene Expression in Gilthead Sea Bream (*Sparus aurata*) Juveniles: Insights in Fish Feeds for Increased Circularity and Resource Utilization

**DOI:** 10.3389/fphys.2021.748265

**Published:** 2021-10-05

**Authors:** Fernando Naya-Català, Gabriella do Vale Pereira, M. Carla Piazzon, Ana Margarida Fernandes, Josep Alvar Calduch-Giner, Ariadna Sitjà-Bobadilla, Luis E. C. Conceição, Jaume Pérez-Sánchez

**Affiliations:** ^1^Nutrigenomics and Fish Growth Endocrinology Group, Institute of Aquaculture Torre de la Sal (IATS-CSIC), Castellón, Spain; ^2^SPAROS Lda, Area Empresarial de Marim, Olhăo, Portugal; ^3^Fish Pathology Group, Institute of Aquaculture Torre de la Sal (IATS-CSIC), Castellón, Spain; ^4^Faculty of Biosciences and Aquaculture, Nord University, Bodø, Norway

**Keywords:** fish meal, processed animal proteins, insect proteins, algae meal, gut microbiota, host transcriptomics, inflammatory markers, lipid metabolism

## Abstract

New types of fish feed based on processed animal proteins (PAPs), insect meal, yeast, and microbial biomasses have been used with success in gilthead sea bream. However, some drawback effects on feed conversion and inflammatory systemic markers were reported in different degrees with PAP- and non-PAP-based feed formulations. Here, we focused on the effects of control and two experimental diets on gut mucosal-adherent microbiota, and how it correlated with host transcriptomics at the local (intestine) and systemic (liver and head kidney) levels. The use of tissue-specific PCR-arrays of 93 genes in total rendered 13, 12, and 9 differentially expressed (DE) genes in the intestine, liver, and head kidney, respectively. Illumina sequencing of gut microbiota yielded a mean of 125,350 reads per sample, assigned to 1,281 operational taxonomic unit (OTUs). Bacterial richness and alpha diversity were lower in fish fed with the PAP diet, and discriminant analysis displayed 135 OTUs driving the separation between groups with 43 taxa correlating with 27 DE genes. The highest expression of intestinal *pcna* and *alpi* was achieved in PAP fish with intermediate values in non-PAP, being the pro-inflammatory action of *alpi* associated with the presence of *Psychrobacter piscatorii*. The intestinal *muc13* gene was down-regulated in non-PAP fish, with this gene being negatively correlated with anaerobic (Chloroflexi and *Anoxybacillus*) and metal-reducing (*Pelosinus* and *Psychrosinus*) bacteria. Other inflammatory markers (*igm, il8, tnf*α) were up-regulated in PAP fish, positively correlating the intestinal *igm* gene with the inflammasome activator *Escherichia/Shigella*, whereas the systemic expression of *il8* and *tnf*α was negatively correlated with the Bacilli class in PAP fish and positively correlated with *Paracoccus yeei* in non-PAP fish. Overall changes in the expression pattern of *il10*, galectins (*lgals1, lgals8*), and toll-like receptors (*tlr2, tlr5, tlr9*) reinforced the anti-inflammatory profile of fish fed with the non-PAP diet, with these gene markers being associated with a wide range of OTUs. A gut microbiota-liver axis was also established, linking the microbial generation of short chain fatty acids with the fueling of *scd1*- and *elovl6*-mediated lipogenesis. In summary, by correlating the microbiome with host gene expression, we offer new insights in the evaluation of fish diets promoting gut and metabolism homeostasis, and ultimately, the health of farmed fish.

## Introduction

Fish meal (FM) is the gold dietary protein in aquafeeds (Tacon and Metian, 2015; Ytrestøyl et al., 2015), but the global increase in aquaculture production needs to be supported by alternative feed ingredients, which very often, have a negative impact on growth, intestinal health, and immuno-competence of farmed marine fish (Conceição et al., 2012; Krogdahl et al., 2015; Estensoro et al., 2016; Aragão et al., 2020). Traditionally, plant proteins have been considered as the most obvious FM alternative, but high levels of replacement can induce different signs of enteritis, including the shortening of mucosal folds, thickening of the lamina propia and submucosa, and infiltration of the distal intestine by inflammatory cells (Urán et al., 2009; Romarheim et al., 2013, Booman et al., 2018). These drawback effects are differentially exacerbated in each farmed fish species, but the supplementation of plant-based diets with fish protein hydrolysates or short-chain fatty acids (SCFAs) helps to mitigate most gut health detrimental effects in salmonid and non-salmonid fish (Estensoro et al., 2016; Piazzon et al., 2017; Egerton et al., 2020). However, novel ingredients and formulations need to be investigated to produce improved, efficient, and sustainable fish aquafeeds.

Seaweed polysaccharides have the capacity to regulate the non-specific host immunity, and supplementation of practical diets, with *Gracilaria* sp. improving the resistance of European sea bass (*Dicentrarchus labrax*) to *Photobacterium damselae* (Peixoto et al., 2019). In the same line, antioxidant extracts of *Gracilaria* protected against oxidative stress, improving the stress resilience of gilthead sea bream during acute hypoxia (Magnoni et al., 2017). Moreover, the supplementation of FM-free diets with marine diatomeas (*Phaeodactylum tricornutum*) stimulated the immune system of gilthead sea bream, which might be relevant as a prophylactic measure before predictable stressful events (Reis et al., 2021). Additionally, algae or algae-based products are currently used to replace or reduce the inclusion level of fish oil (FO) in aquafeeds, assuring the dietary supply of n-3 polyunsaturated fatty acids to meet the nutritional requirements of fish, as well as the healthy value of seafood products for human consumption (Maldonado-Othón et al., 2020; Pereira et al., 2020).

Processed animal proteins (PAPs), such as poultry meal, blood meal, and feather meal, are also used as a replacement for FM in practical fish diets (Wu et al., 2018; Ferrer Llagostera et al., 2019; Solé-Jiménez et al., 2021). Likewise, insect meal products, such as black soldier fly meal and defatted *Tenebrio* meal, did not compromise gut health and fish performance (Welker et al., 2007; Sánchez-Muros et al., 2016; Magalhães et al., 2017; Basto et al., 2021) and in some cases, even improved performance. Microbial biomasses have also been proposed as an effective replacement for marine feedstuffs in fish and shrimp diets (Delamare-Deboutteville et al., 2019; Simon et al., 2019, 2020). Such new and emerging ingredients have, in fact, been on the agenda of formulators for some time, but they are still available in low amounts, and their costs are often too high in comparison with most conventional feed ingredients. Moreover, many of these ingredients have been tested on a one-by-one basis rather than with different formulation combinations, although by this way important advances were achieved. Thus, a recent study on gilthead sea bream pointed out that sustainable PAP- and non-PAP-based feed formulations are able to support high growth rates when theoretical nutrient requirements are met by the diet (Fernandes et al., 2021). However, the use of PAP-based feed formulations was related to a slight impairment in feed conversion ratio (FCR) in association with a progressive down-regulated expression of hepatic insulin-like growth factor-I, a well-known marker of growth performance in fish, gilthead sea bream in particular (Pérez-Sánchez et al., 2018). Additionally, fish fed with the highest amount of PAP feed ingredients showed a pro-inflammatory status, supported by the up-regulated expression in the head kidney of several cytokines (*il1*β, *tnf*α), chemokines (*ck8*), and T-cell markers (*cd3*ζ, *cd4-1, cd8*α).

In this study, we focused on gut health indicators using samples from the study of Fernandes et al. (2021) to address the main effects of PAP- and non-PAP feed formulations on the composition of mucosal adherent bacteria from the anterior intestine (AI) in association with changes in the host transcriptomic profile of the intestine liver, and head kidney. Over the course of the last 5 years, the number of studies on fish, gilthead sea bream in particular, addressing the nutritional regulation of gut microbiota has increased exponentially (e.g., Piazzon et al., 2017, 2020; Rimoldi et al., 2019, 2020; Firmino et al., 2021; Moroni et al., 2021; Pelusio et al., 2021; Solé-Jiménez et al., 2021). Although our study did not include probiotics, previous studies on fish supported the gut-to-brain communication, and interestingly, the dietary administration of *Lactobacillus rhamnosus* interfered in shoaling behavior and brain expression levels of genes involved in serotonin signaling and metabolism (Borrelli et al., 2016). Recently, significant differences in the intestinal expression pattern of key genes involved in innate and acquired immunity were reported when gilthead sea bream juveniles fed with probiotic (*Lactococcus lactis*) and control diets (Moroni et al., 2021) were compared. However, to our knowledge, this is the first report to link fish alternative feed formulations with changes in gut microbiota and host transcriptomics at the local and/or systemic level in typical farmed marine fish.

## Materials and Methods

### Ethics

The feeding trial was conducted by trained scientists (following the Federation of European Laboratory Animal Science Associations, FELASA, category C recommendations), according to the animal experimentation guidelines on the protection of animals used for scientific purposes from the European directive 2010/63/UE at the experimental facilities of RIASEARCH/SPAROS (Murtosa, Portugal).

### Diets

Three diets were formulated and manufactured by SPAROS Lda (Olhăo, Portugal) according to the known nutritional requirements for gilthead sea bream (NRC 2011) (Table 1). The control diet (CRTL) contained 20% FM and high levels of traditional vegetable proteins following current industry formulations. Both the PAP- and non-PAP-based feed formulations contained insect meal, fish by-products, and microbial and yeast biomasses as replacements for FM and vegetable protein sources. The PAP-based diet also comprised poultry meal, feather meal hydrolysate, and porcine blood meal as main protein sources. Alternatively, the non-PAP-based diet included *Spirulina* and *Chlorella* meals as novel protein sources in fish aquafeeds. FO was replaced in both the PAP and NoPAP diets by an aquaculture by-product (salmon oil), DHA-rich algae biomass (*Schizochytrium*), and rapeseed oil. Additionally, both diets were adequately supplemented with rapeseed lecithin, phosphorous, L-tryptophan, DL-methionine, and L-taurine.

**Table 1:** Ingredients and chemical composition of the experimental diets.

**Ingredient (%)**	**CTRL**	**NoPAP**	**PAP**
Fishmeal LT70	20		
Fishmeal 60 (by-products)		5	
Fish hydrolysate (by-products)		5	5
Insect meal		5	5
Microbial protein meal		5	5
Yeast protein meal		2.5	2.5
Feather meal hydrolysate			5
Porcine blood meal			3
Poultry meal 65	5		20
Microalgae meal (*Spirulina*)		5	
Microalgae meal (*Chlorella*)		0.5	
Soy protein concentrate	9		
Pea protein concentrate		4.1	
Wheat gluten	4	4	
Corn gluten meal	10	15	4.5
Soybean meal 48	12		
Rapeseed meal	4	11.5	5.7
Wheat meal	8.47		
Pea starch	3	7.9	6
Yellow peas	6.2	3	14.58
Fish oil	6		
Salmon oil		3	3
DHA-rich algae (*Schizochytrium*)		3.2	3.6
Rapeseed oil	8.26	8.5	6
Rapeseed lecithin	0.6	1	1
Vitamin and mineral premix	1	1	1
Vitamin C (35%)	0.1	0.1	0.1
Brewer's yeast		4	4
Macroalgae MIX		2	2
Antioxidant	0.2	0.2	0.2
Sodium propionate	0.1	0.1	0.1
Monocalcium phosphate	2	2.5	1.9
L-Tryptophan	0.05	0.18	0.15
DL-Methionine		0.2	0.15
L-Taurine		0.5	0.5
Yttrium oxide	0.02	0.02	0.02
*Chemical Composition*			
Crude Protein (%)	44.2	44.88	44.71
Crude Lipid (%)	17.84	17.62	16.29
EPA +DHA (%)	1.9	1.5	1.5

### Experimental Setup and Sampling

A total of 880 juveniles of gilthead sea bream (initial body weight 55–56 g on average) from a commercial hatchery (Sonrionansa, Santander, Spain) were reared at RIASEARCH/SPAROS trial facilities (Murtosa, Portugal) with the CTRL and experimental diets as reported in Fernandes et al. (2021). In short, the trial was run for 77 days in replicated 500-L tanks, in a flow-through system with brackish water (18 ppt) and constant photoperiod (12 h ligh:12 h dark). During that time, the fish were hand-fed *ad libitum* to satiation three times a day, water temperature was 22.03 ± 1.4°C, and water oxygen concentration remained higher than 5.5 mg/L.

At the end of the trial, 48-h fasted fish were sampled for both gut bacterial microbiota and gene expression analyses (nine fish per feed formulation from three randomly selected tanks, three fish per tank). All the fish were euthanised by overdose of anesthetic 3-aminobezoic acid ethyl ester (MS-222, 0.1 g/L), according to the good practices of fish health and welfare. The skin surface of the abdomen was cleaned with ethanol 70%, and a cut from the anus to the esophagus was made to remove the intestine, which was sampled and placed on a sterile Petri dish. Tissue portions (~0.4 cm) of the anterior intestine (AI; immediately after the pyloric caeca) were put in RNA later for subsequent gene expression analysis. The remaining part of the AI was then opened and washed with phosphate-buffered saline (PBS) to remove non-adherent materials and bacteria. The tissue was transferred to a clean Petri dish, and the internal mucus was scraped out with the blunt end of a sterile scalpel. The sampled mucus was then placed into sterile cryotubes and stored at −80°C until bacterial DNA extraction for microbiota analysis using the High Pure PCR Template Preparation Kit (Sigma-Aldrich, St. Louis, MO, United States) and including a lysozyme lysis step, as previously described (Piazzon et al., 2019). In this study, the AI was used because of its importance in nutrient absorption and metabolism. The procedure targeted mucosa-colonizing autochthonous bacteria, which have a more direct impact on fish physiology. Allochthonous bacteria (not resident) cannot colonize these habitats under normal conditions and have a more transient impact on the host (Hao and Lee, 2004). The 48-h fasting period was chosen to ensure sample stability and avoid contamination by allochthonous bacteria, which are more difficult to eliminate when the intestines are filled with fecal matter. We are aware that fasting can affect intestinal microbiota (Xia et al., 2014; Nebo et al., 2017; Mekuchi et al., 2018), but these effects have been described for longer fasting periods or allochthonous populations, whereas autochthonous populations are more stable. In any case, we followed the standard procedure used in other gilthead sea bream gene expression and microbiota studies (Estensoro et al., 2016; Piazzon et al., 2019, 2020; Solé-Jiménez et al., 2021), allowing for comparisons to be made.

### Illumina MiSeq Sequencing and Bioinformatics Analysis

The V3-V4 region of the 16S rRNA gene (reference nucleotide interval 341-805 nt) was sequenced using the Illumina MiSeq System (Illumina, San Diego, CA, United States) (2 × 300 paired-end run) at the Genomics Unit from the Madrid Science Park Foundation (FPCM). The details on the PCR and sequencing of amplicons are described elsewhere (Piazzon et al., 2019). Regarding bioinformatics analysis, raw forward and reverse reads were quality-filtered using FastQC (http://www.bioinformatics.babraham.ac.uk/projects/fastqc/) and pre-processed using Prinseq (Schmieder and Edwards, 2011). Terminal N bases were trimmed in both ends, and sequences with >5% of total N bases were discarded. Reads that were <150-bp long, with Phred quality score <28 in both of the sequence ends, and with a Phred average quality score <26 were excluded. Forward and reverse reads were merged using VSEARCH (Rognes et al., 2016). Raw sequence data from six samples did not display enough quality standards to be included in the analysis and were removed from further analysis. The remaining 21 samples (7 from the PAP, 9 from non-PAP, and 5 from CTRL groups) were uploaded to the Sequence Read Archive (SRA) under Bioproject accession number PRJNA745265 (BioSample accession numbers: SAMN20157689-709).

Bacteria taxonomy assignment was performed using the Ribosomal Database Project (RDP) release 11 as a reference database (Cole et al., 2014). The reads were aligned with a custom-made pipeline using VSEARCH and BLAST (Altschul et al., 1990; Rognes et al., 2016). Alignment was performed establishing high stringency filters (≥90% sequence identity, ≥90% query coverage). Taxonomic assignment results were filtered, and data were summarized in an operational taxonomic units (OTUs) table. Sample depths were normalized by total sum scaling and then made proportional to the total sequencing depth following the recommendations described by McKnight et al. (2019).

### Inferred Metagenome and Pathway Analysis

Piphillin was used to normalize the amplicon data with 16S rRNA gene copy number and to infer metagenomic contents (Iwai et al., 2016). This analysis was performed using only the OTUs that significantly drove the separation by diet in the supervised partial least-squares discriminant analysis (PLS-DA) (see Statistics section). For the analysis, a sequence identity cut-off of 97% was implemented, and the inferred metagenomic functions were assigned using the Kyoto Encyclopedia of Genes and Genomes (KEGG, Oct 2018 Release) database. The raw KEGG pathway output from Piphillin was analyzed with the R Bioconductor package DESeq2 using default parameters after fractional counts were floored to the nearest integer (Love et al., 2014; Bledsoe et al., 2016; Piazzon et al., 2020).

### Gene Expression

Total RNA from AI (9 fish/diet) was extracted using a MagMax-96 total RNA isolation kit (Life Technologies, Carlsbad, CA, United States). The RNA yield per sample was higher than 3.5 μg with absorbance measures (A260/280) of 1.9–2.2. Complementary DNA (cDNA) was synthesized with the High-Capacity cDNA Archive Kit (Applied Biosystems, Foster City, CA, United States), using random decamers and 500 ng of total RNA in a final volume of 100 μl. Reverse transcription (RT) reactions were incubated for 10 min at 25°C and for 2 h at 37°C. Negative control reactions were run without the enzyme. As reported elsewhere (Estensoro et al., 2016), a customized PCR array layout was designed for the simultaneous profiling of a panel of 43 selected genes, including markers of epithelial integrity (11), nutrient transport (3), mucins (3), cytokines (9), immunoglobulins (2), cell markers, chemokines, and chemokine receptors (7), and pattern recognition receptors (PRRs) (8) (Table 2). Quantitative PCR (qPCR) reactions were performed using the iCycler IQ Real-Time Detection System (Bio-Rad, Hercules, CA, United States). Diluted RT reactions (× 6) were used for qPCR assays in a 25-μl volume, in combination with SYBR Green Master Mix (Bio-Rad, Hercules, CA, United States) and specific primers at a final concentration of 0.9 μM (Supplementary Table 1). The program used for PCR amplification included an initial denaturation step at 95°C for 3 min, followed by 40 cycles of denaturation for 15 s at 95°C, and annealing/extension for 60 s at 60°C. All the pipetting operations were executed by means of an EpMotion 5070 Liquid Handling Robot (Eppendorf, Hamburg, Germany) to improve data reproducibility. The efficiency of PCRs (>92%) was checked, and the specificity of reactions was verified by analyzing the melting curves (ramping rates of 0.5°C/10 s over a temperature range of 55–95°C) and linearity of serial dilutions of RT reactions (*r*^2^ > 0.98). Fluorescence data acquired during the extension phase were normalized with the delta-delta CT method (Livak and Schmittgen, 2001), using beta-actin as a housekeeping gene because of its stability under different experimental conditions (average CT between experimental groups varied less than 0.2).

**Table 2:** Polymerase chain reaction (PCR)-array layout for intestine gene expression profiling in sea bream.

**Function**	**Gene**	**Symbol**	**GenBank**
Epithelial integrity	Proliferating cell nuclear antigen	*pcna*	KF857335
	Transcription factor HES-1-B	*hes1-b*	KF857344
	Krueppel-like factor 4	*klf4*	KF857346
	Claudin-12	*cldn12*	KF861992
	Claudin-15	*cldn15*	KF861993
	Cadherin-1	*cdh1*	KF861995
	Cadherin-17	*cdh17*	KF861996
	Tight junction protein ZO-1	*tjp1*	KF861994
	Desmoplakin	*dsp*	KF861999
	Gap junction Cx32.2 protein	*cx32.2*	KF862000
	Coxsackievirus and adenovirus receptor homolog	*cxadr*	KF861998
Nutrient transport	Intestinal-type alkaline phosphatase	*alpi*	KF857309
	Liver type fatty acid-binding protein	*fabp1*	KF857311
	Intestinal fatty acid-binding protein	*fabp2*	KF857310
Mucus production	Mucin 2	*muc2*	JQ277710
	Mucin 13	*muc13*	JQ277713
	Intestinal mucin	*i-muc*	JQ277712
Cytokines	Tumor necrosis factor-alpha	*tnfα*	AJ413189
	Interleukin-1 beta	*il1β*	AJ419178
	Interleukin-6	*il6*	EU244588
	Interleukin-7	*il7*	JX976618
	Interleukin-8	*il8*	JX976619
	Interleukin-10	*il10*	JX976621
	Interleukin-12 subunit beta	*il12*	JX976624
	Interleukin-15	*il15*	JX976625
	Interleukin-34	*il34*	JX976629
Cell markers, chemokines and chemokine receptors	Cluster of differentiation 4-1	*cd4-1*	AM489485
	Cluster of differentiation 8 beta	*cd8β*	KX231275
	C-C chemokine receptor type 3	*ccr3*	KF857317
	C-C chemokine receptor type 9	*ccr9*	KF857318
	C-C chemokine receptor type 11	*ccr11*	KF857319
	C-C chemokine CK8 / C-C motif chemokine 20	*ck8/ cl20*	GU181393
	Macrophage colony-stimulating factor 1 receptor 1	*csf1r1*	AM050293
Immunoglobulins	Immunoglobulin M	*igm*	JQ811851
	Immunoglobulin T	*igt*	KX599201
Pattern recognition receptors (PRRs)	Galectin-1	*lgals1*	KF862003
	Galectin-8	*lgals8*	KF862004
	Toll-like receptor 2	*tlr2*	KF857323
	Toll-like receptor 5	*tlr5*	KF857324
	Toll-like receptor 9	*tlr9*	AY751797
	C-type lectin domain family 10 member A	*clec10a*	KF857329
	Macrophage mannose receptor 1	*mrc1*	KF857326
	Fucolectin	*fcl*	KF857331

### Statistical Analysis

Biometric and gene expression data were analyzed by one-way ANOVA using the R stats package and SigmaPlot v.14.5 (Systat Software Inc., San Jose, CA, United States). The data have been checked previously for normal distribution (Shapiro–Wilk test) and homogeneity of variances (F test). Following ANOVA, if appropriate, multiple comparisons between fish groups were computed by Dunn's post-test. The statistical significance of growth parameters was tested at *P* < 0.05, whereas the threshold for differentially expressed (DE) genes was established at *P* < 0.1. Fish specimens sampled and used for gene expression analyses (nine fish/diet) were the same as those used in Fernandes et al. (2021) and in 16S sequencing (nine fish/diet before quality filter). Rarefaction curves (observed taxonomic assignations vs. number of sequences), species richness estimates, and alpha diversity indexes were obtained using the R package phyloseq (McMurdie and Holmes, 2013). To determine the coverage for microbial communities, the ratio between observed and expected OTUs (determined by the Chao1 index) was calculated. Differences in species richness, diversity indexes, and phylum abundance were determined by Kruskal–Wallis test followed by Dunn's post-test, with a significance threshold of *P* < 0.05. Beta diversity across groups was tested by permutational multivariate ANOVA (PERMANOVA), using the non-parametric method *adonis* from the Vegan R package with 10,000 random permutations.

To study the separation among the groups, supervised PLS-DA and hierarchical clustering of samples were sequentially performed using EZinfo v3.0 (Umetrics, Umeå, Sweden) and R package ggplot2, respectively. Values of normalized counts of OTUs present in five or more of the samples were included in the analyses. The contribution of the different genes to the group separation was determined by the minimum variable importance in the projection (VIP) values achieving the complete clustering of the conditions with a VIP value ≥ 1, considered to be an adequate threshold to determine discriminant variables in the PLS-DA model (Wold et al., 2001; Li et al., 2012; Kieffer et al., 2016). Hotelling's T2 statistic was calculated with the multivariate software package Ezinfo v3.0 to detect outliers in the model and reported. The quality of the PLS-DA model was evaluated by the parameters R2Y (cum) and Q2 (cum), which indicate fit and prediction ability, respectively. To assess whether the supervised model was being over-fitted, a validation test consisting of 500 random permutations was performed using the Bioconductor R package ropls (Thévenot et al., 2015). In order to determine the OTUs most likely to explain differences between and among the feed formulations, a linear discriminant analysis (LDA) effect size (LEfSe) method (Segata et al., 2011) was used with the online tool Galaxy v1.2 (Afgan et al., 2018). OTUs with VIP ≥ 1 were included in this analysis, and statistically significant differences were retrieved by the factorial Kruskal–Wallis test, followed by the pairwise Wilcoxon post-test with a significance threshold of α = 0.05.

The inferred metagenomics pathways were considered differentially represented using an FDR-corrected significance threshold of 0.05. For the correlation analysis, only 16S sequencing samples that passed the quality filter and remained after the T2 Hotelling outlier test (20 samples in total; 9 from non-PAP, 7 from PAP, 4 from CTRL) were included. The outliers were excluded from further analysis. Spearman correlation was calculated between the normalized values of discriminant OTUs abundances in the 20 samples and normalized values of DE gene expression in its corresponding sampled specimen (Weiss et al., 2016). The corresponding *P*-values were calculated using the *cor.test* function of the corrplot R package, with a two-sided alternative hypothesis. Significant gene-OTU correlations were considered at *P* < 0.01, and visualiszed with the corrplots R package and Cytoscape v3.8.2 (Smoot et al., 2011). For the same fish, the correlation analysis was also performed for the DE genes of the liver (12) and head-kidney (9), retrieved from the study of Fernandes et al. (2021).

## Results

### Growth Performance

Data on body weight, feed intake, and FCR from the whole population are taken from Fernandes et al. (2021), and reported in Supplementary Table 2. These parameters did not change significantly (*P* < 0.05) between CTRL and fish fed with the NoPAP diet. The equality was less evident for fish fed with the PAP diet, and slight changes in weight gain and feed intake resulted in a statistically significant increase in FCR. Concerning the individuals sampled for microbiota and gene expression analyses, the recorded body weight did not deviate from the expected values, with the trend being lowest in the body weight of fish fed with the PAP-based diet.

### Alpha Diversity and Microbiota Composition

A total of 2,632,361 high-quality reads from the 21 sequenced samples were assigned to 1,281 OTUs at a 97% identity threshold, ranging from 70,301 to 164,133 reads per sample (in average 125,350 reads per sample) (Supplementary Table 3). Rarefaction curves approximated saturation, showing an optimal number of sequences obtained for the analysis (Supplementary Figure 1). Results of the coverage ratio in terms of richness also confirmed the adequateness of the samples, with an average value of 61.44%. Out of the 1,281 OTUs, 84.6% was classified up to the level of species, 93.2% to the level of genus, 97.4% to the level of family, 98.7% to the level of order, 99.4% to the level of class, and 99.9% to the level of phylum.

**Figure 1:**
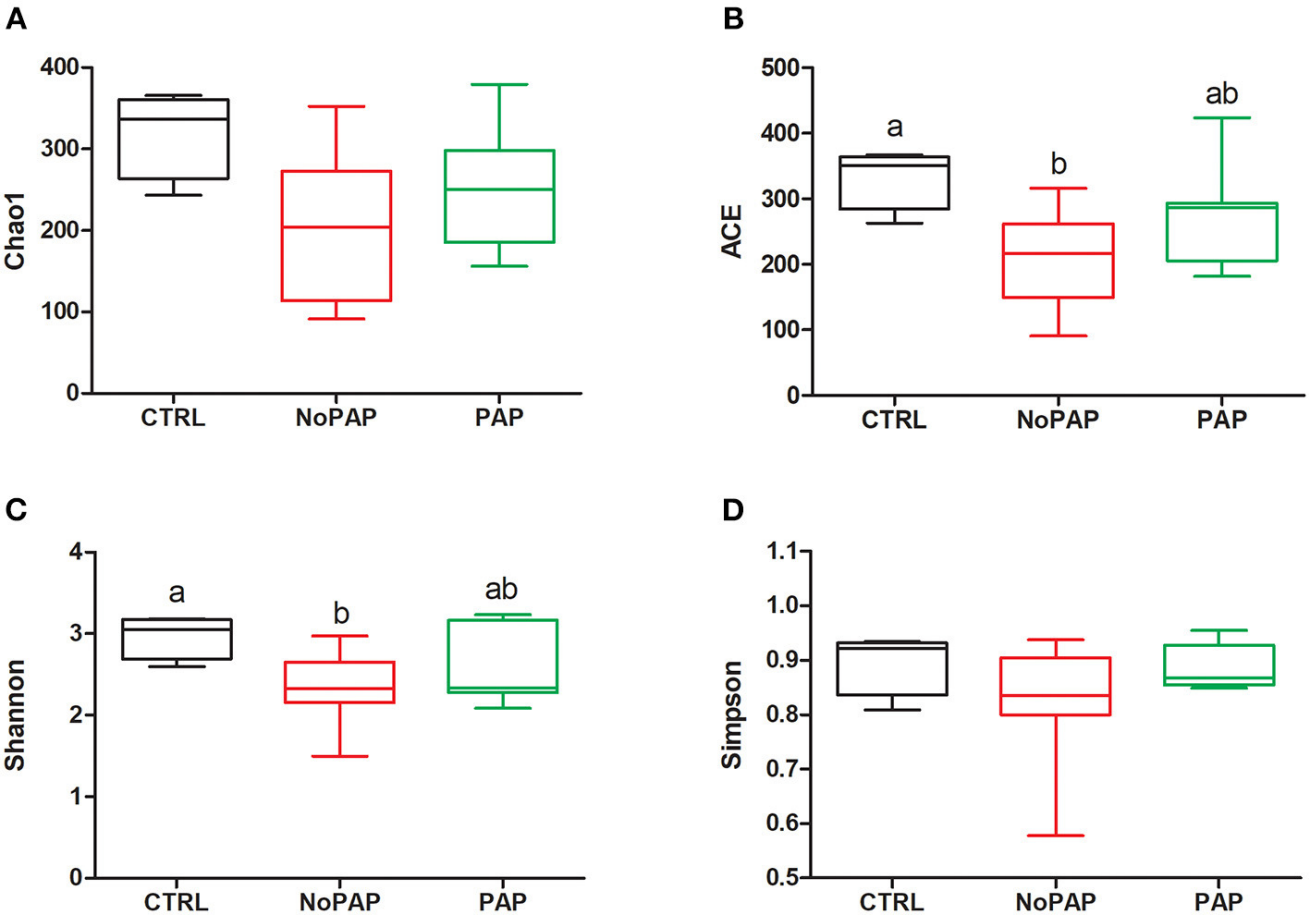
Box plots representing the mean (min-max) of richness estimates [**(A)** Chao1 and **(B)** ACE] and diversity indexes [**(C)** Shannon and **(D)** Simpson] of the intestinal microbial populations found in fish fed with the CTRL (*n* = 4), non-PAP (*n* = 9), and PAP (*n* = 7) diets. Different letters indicate significant differences among the groups (Kruskal–Wallis with Dunn's post-test, *P* < 0.05).

**Figure 2:**
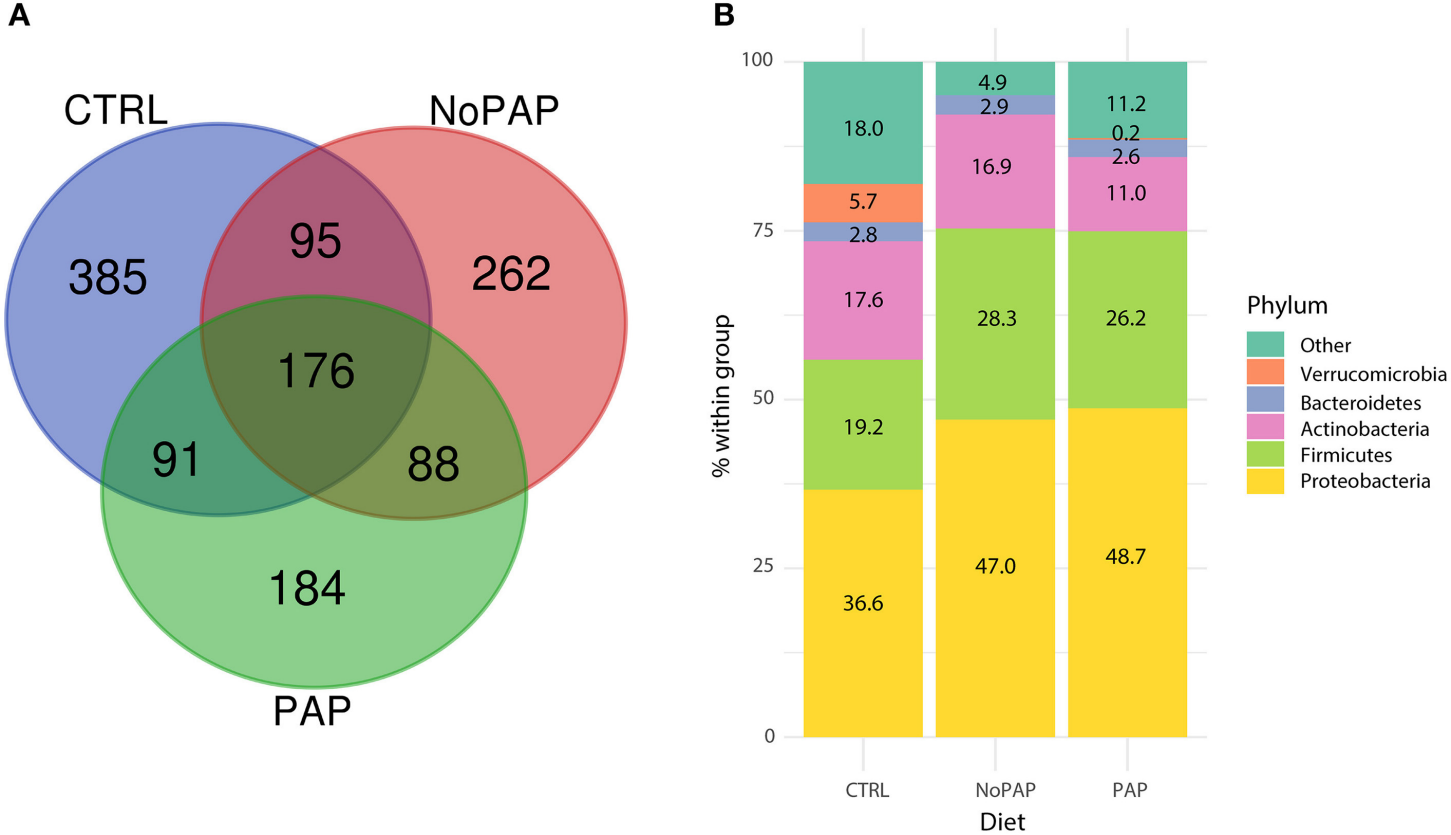
**(A)** Venn diagram showing unique and shared operational taxonomic units (OTUs) in the intestines of fish fed with the three experimental diets. The 176 common OTUs represent 63.5, 69.4, and 60.5% of the overall microbiota in the CTRL, non-PAP, and PAP groups, respectively. Unique OTUs for the CTRL, non-PAP, and PAP groups represent 16.25, 11.8, and 7.3% of the overall bacterial composition, respectively. **(B)** Stacked bar chart representing the relative abundance of bacterial phyla in the three dietary groups. Only the phyla that are present in at least 1% in one of the groups are represented. No significant differences were found among the groups (Kruskal–Wallis + Holm–Sidak tests, *P* > 0.05).

Figure 1 shows that the non-PAP diet induced a significant decrease in richness and alpha diversity in comparison with the CTRL group, when the ACE and Shannon estimators (*P* < 0.05), respectively, were considered. A total of 747, 621, and 539 OTUs were assigned to the CTRL, non-PAP, and PAP fish (Figure 2A). From them, 176 OTUs were present in all the dietary groups, representing more than 60% of the overall bacterial composition in all the groups, whereas 385 (16.2% of the total microbiota), 262 (11.8%), and 184 (7.3%) were present exclusively in the CTRL, non-PAP, and PAP fish, respectively. No significant differences among the groups (Kruskal–Wallis test, followed by Dunn's post-test, *P* < 0.05) were detected when taxonomic assignations were collapsed to the phylum level (Figure 2B). Proteobacteria was the most abundant phylum, reaching values from 35 to 50% of the total bacterial composition, followed by Firmicutes (19–29%), Actinobacteria (11–18%), and Bacteroidetes (2.5–3%). Verrucomicrobia was relatively abundant in the control diet (5.7%), but decreased in the non-PAP and PAP fish (<0.5%). The most abundant genera (>1% in at least one dietary group) are depicted in Supplementary Figure 2.

### Beta Diversity and Discriminant Analyses

**Figure 3:**
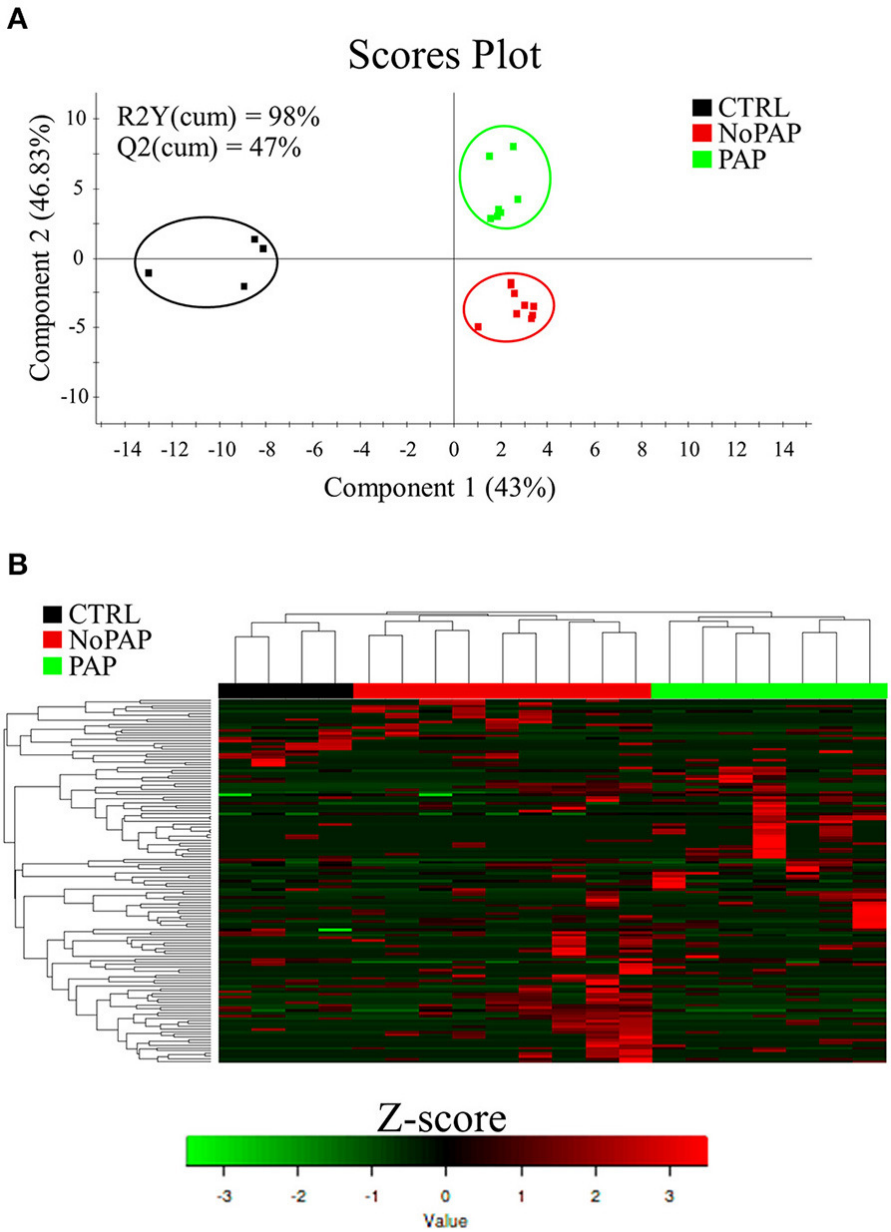
**(A)** Two-dimensional partial least-squares discriminant analysis (PLS-DA) score plot constructed using the variable diet representing the distribution of the samples between the first two components in the model. The validation by permutation test can be found in Supplementary Figure 3. **(B)** Heatmap representing the abundance distribution (Z-score) of the OTUs identified to drive the separation by diet (VIP ≥ 1).

Regarding beta diversity, statistically significant differences among the dietary groups were found (PERMANOVA *P* = 0.049, *F* = 1.0514, *R*^2^ = 0.1101). To further evaluate differences in the bacterial composition among the groups, a partial least squares discriminant analysis (PLS-DA) was performed. The discriminant model was based on four components, which explained 98% [R2Y(cum)] and predicted 47% [Q2Y(cum)] of the total variance (Figure 3A). During the statistical processing to construct the model, one fish from the CTRL group appeared as an outlier and was excluded from the model. The fit of the resulting PLS-DA model was validated by a permutation test (Supplementary Figure 3). The final model clearly separated the CTRL from the NoPAP and PAP fish in the first component (43% explained variance), whereas the second component mainly separated the fish fed with the PAP diet from the other two groups (>46% explained variance). According to this, the hierarchical clustering grouped together the CTRL and PAP fed fish, and all the samples were properly classified in their respective experimental group (Figure 3B). Filtering by VIP ≥ 1, a total of 135 OTUs mainly drove the separation among the experimental groups. The discriminant OTUs constituted 29.4, 10.6, and 46.5% of the overall microbial composition of fish fed with the CTRL, non-PAP, and PAP diets, respectively (Supplementary Table 4).

The LEfSe analysis revealed a total of 20 strong positive associations (Log_10_LDA Score > 4) between the discriminant OTUs of the PLS-DA analysis and the experimental groups (Supplementary Figures 4A,B). In particular, the analysis revealed that the Pseudomonadaceae family and their genus *Pseudomonas*, as well as *Sphingomonas oligophenolica* and *Paracoccus koreensis*, were more represented in the CTRL group. In the non-PAP fish, a higher representation of the Flavobacteriaceae, Streptococcaceae, and Clostridiaceae families and the *Sphingomonas* and *Staphylococcus* (in particular *S. petrasii*) genera was found. In the PAP fish, the family of Lactobacillales and the *Clostridium* (*C. aciditolerans*), *Maritimibacter* (*M. alkaliphilus*), *Pelosinus*, and *Psychrosinus* genera were overrepresented.

### Inferred Pathways

**Figure 4:**
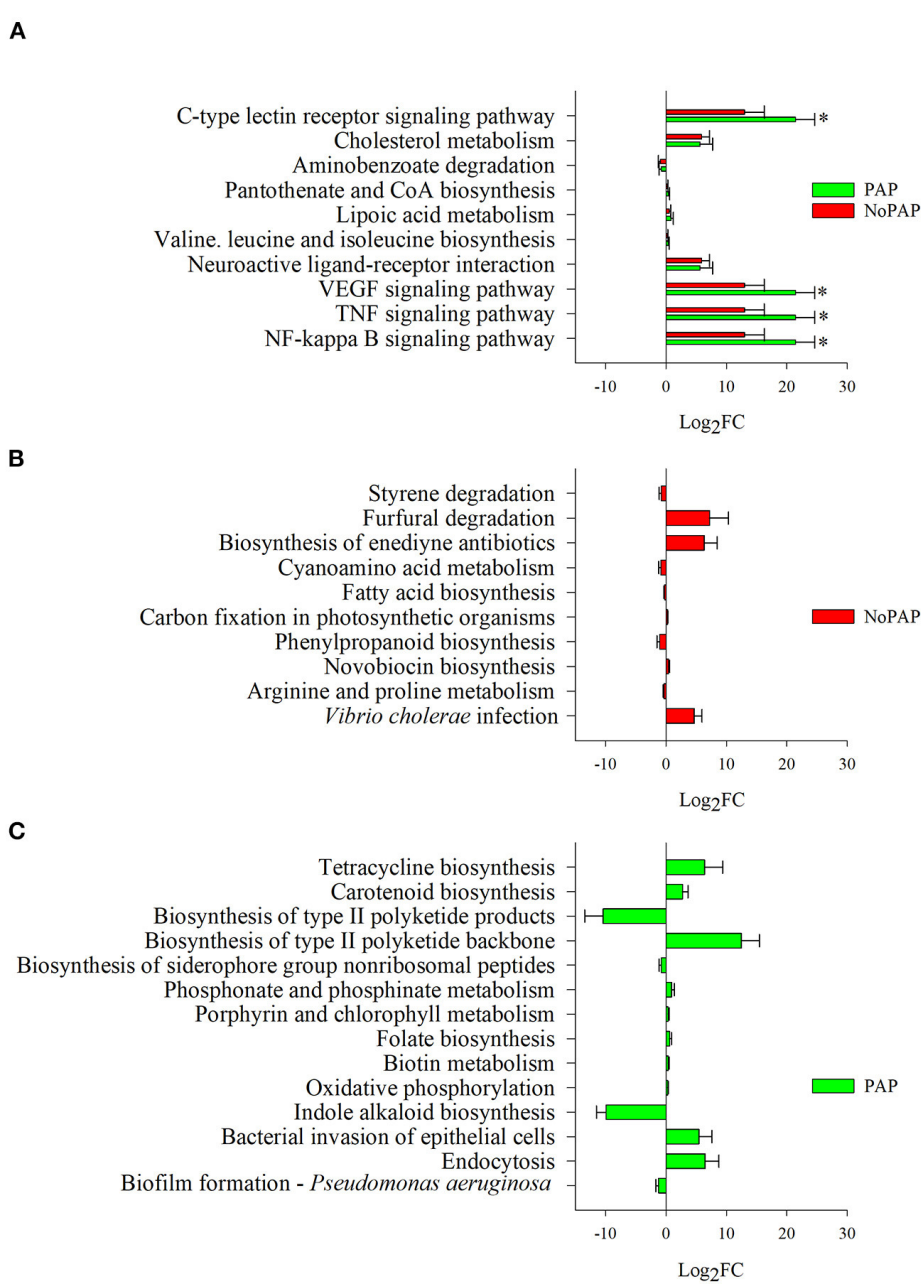
Results from the pathway analysis performed with the predicted metagenome obtained from the discriminant OTUs with VIP ≥ 1. **(A)** Differentially and common represented pathways (padj < 0.05) when comparing the PAP or non-PAP diets with the CTRL diet. The asterisk represents the result of a differential pathway representation (padj < 0.05) between the NoPAP and PAP diets. **(B)** Exclusively differentially represented pathways in the non-PAP vs. CTRL comparison. **(C)** Exclusively differentially represented pathways in the PAP vs. CTRL comparison. Bars show the Log_2_ fold change of differentially over- or under-represented pathways (± standard error of the calculated fold change).

The sequences of the 135 OTUs, driving the separation of dietary groups, were used to discern the potential implication of microbiota in KEGG pathways by an inferred metagenome analysis. The analysis displayed a total of 38 OTUs (VIP ≥ 1) whose genomes were potentially associated with the expression of genes involved in the differentially represented pathways (FDR < 0.05). When compared with the CTRL fish, 20 and 24 pathways showed to be potentially changing in the non-PAP and PAP fish, respectively (Figure 4). Ten of these pathways were common to NoPAP and PAP fish, with an up-regulation of the routes tailoring immune response and inflammation (C-type lectin receptor, VEGF, TNF, and NFκ-B signalling pathways), that was significantly lower (FDR = 0.01) in fish fed the NoPAP diet. Cholesterol metabolism and the neuroactive ligand-receptor interaction pathways were also over-represented to a similar extent in both conditions. Fish fed with the non-PAP and PAP-based feed formulations displayed an exclusive type of response at this level, with the differential representation of 10 and 14 inferred pathways, respectively. The list of bacteria related to each pathway can be found in Supplementary Table 5. This list is obtained from *in silico* inference and only reflects what could be hypothetically occurring, but it is still of value to assess the metabolic capability of bacterial populations.

### Intestinal Gene Expression Profiling

**Figure 5:**
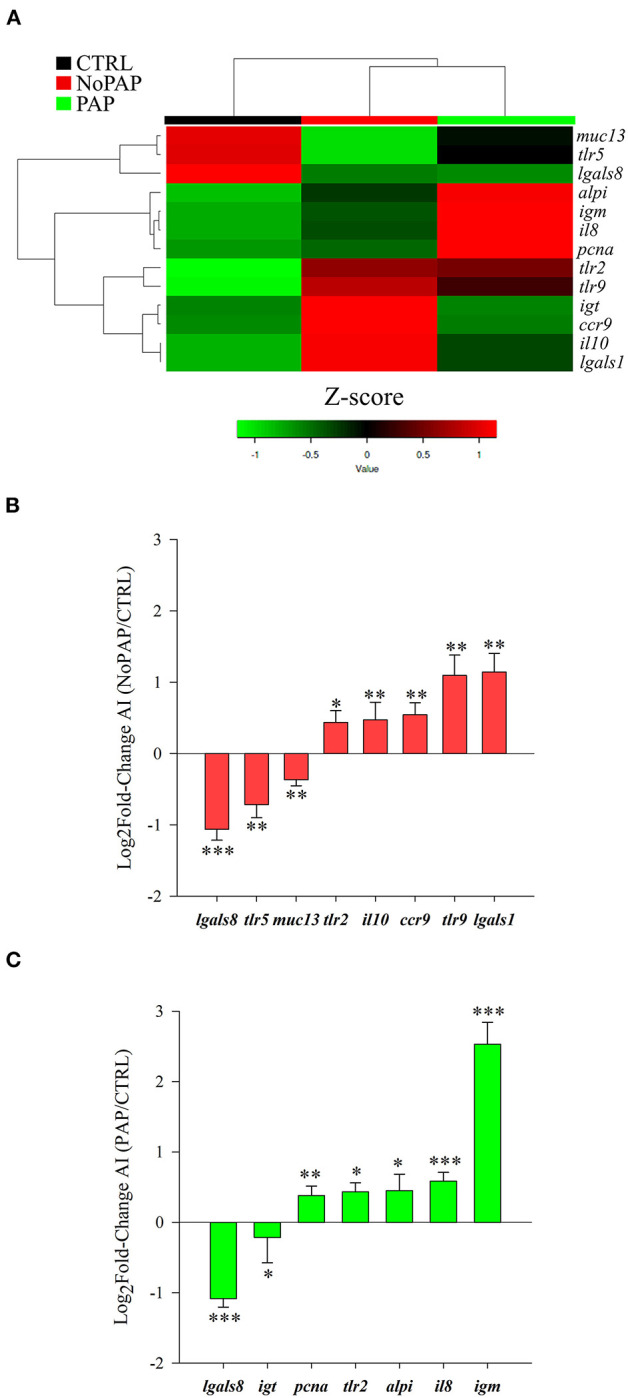
Differentially expressed (DE) genes in the anterior intestine of fish fed with the experimental diets when compared with those fed with the CTRL diet (*P* < 0.05). **(A)** Heatmap depicting the relative expression of the DEGs in the three dietary groups. Log_2_ fold changes of differentially expressed genes (+SEM) in comparisons **(B)** non-PAP vs. CTRL and **(C)** PAP vs. CTRL are shown [asterisks represent statistically significant differences at *P < 0.05, **P < 0.01, and ***P < 0.001].

All the genes included in the intestinal PCR-array were found at detectable levels, and dietary intervention significantly altered the expression pattern of 13 out of 43 genes (Supplementary Table 6), with PAP and non-PAP fish being clustered together in a heatmap expression pattern (Figure 5A). In comparison with the CTRL fish, galectin-8 (*lgals8*) was significantly down-regulated in fish fed with the non-PAP- and PAP-based diets (Figures 5B,C), whereas mucin 13 (*muc13*) and toll-like receptor 5 (*tlr5*) were only down-regulated in the non-PAP fish and immunoglobulin T (*igt*) in the PAP fish (Figures 5B,C). Toll-like receptor 2 (*tlr2*) was up-regulated in both the non-PAP and PAP fish, but the proliferating cell nuclear antigen (*pcna*) was significantly up-regulated in the PAP fish but not in the fish fed with the non-PAP diet (Figures 5B,C). Likewise, interleukin-10 (*il10*), C-C chemokine receptor type 9 (*ccr9*), toll-like receptor 9 (*tlr9*), and galectin-1 (*lgals1*) were up-regulated in the non-PAP fish (Figure 5B). Conversely, the expression of intestinal-type alkaline phosphatase (*alpi*), interleukin-8 (*il8*), and immunoglobulin M (*igm*) was only up-regulated in the fish fed with the PAP diet (Figure 5C).

### Linking Gut Microbial Population and Host Transcriptome

**Figure 6:**
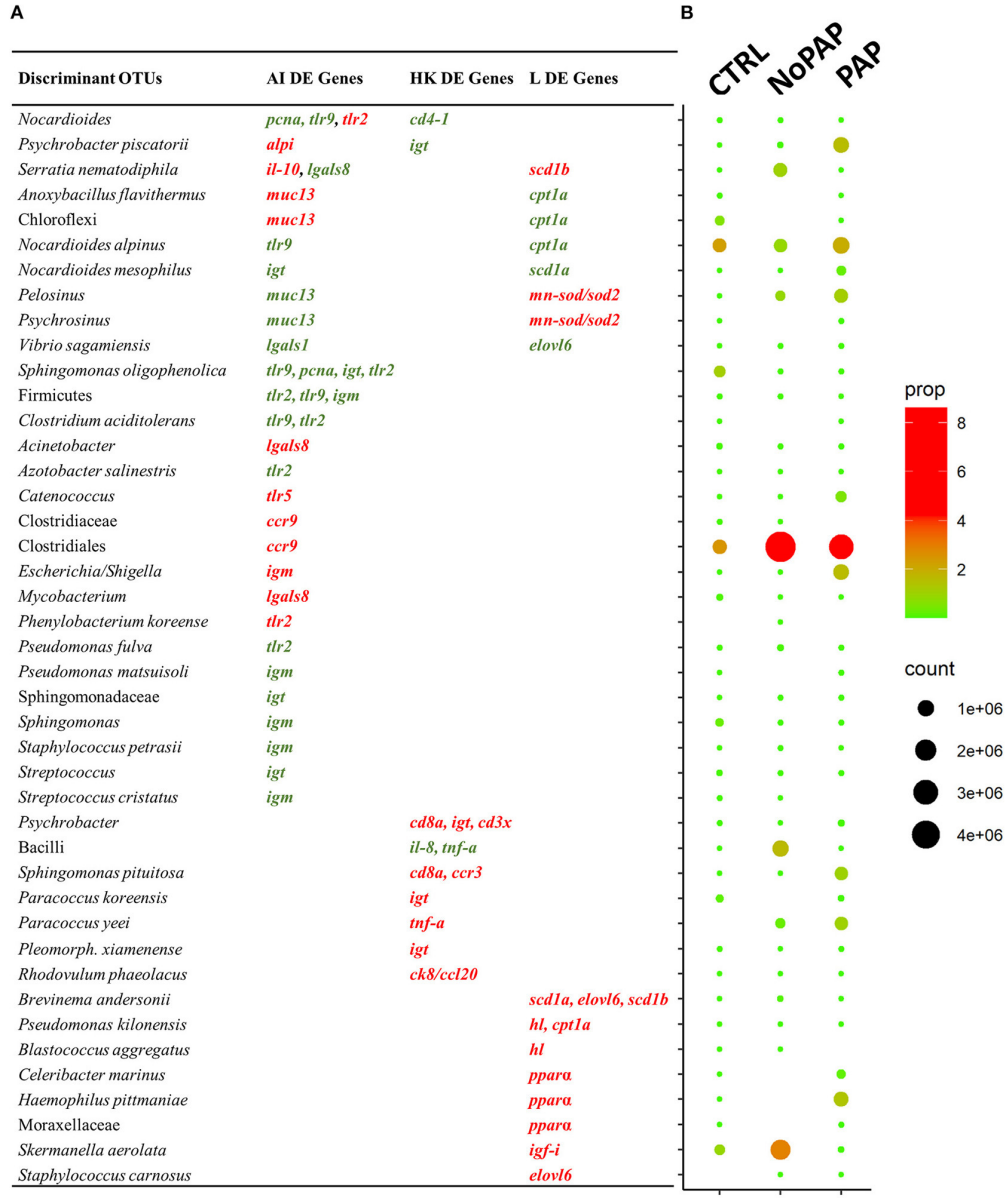
**(A)** List of the 43 discriminant OTUs involved in significant (*P* < 0.01) correlations with intestinal (AI), head kidney (HK), and liver (L) differentially expressed genes (DEGs). Colors of genes in DE gene columns indicate if there is a direct (red) or inverse (green) OTU-gene correlation. **(B)** Dot plot depicting the normalized count number together with the proportion of correlated OTUs among the CTRL and experimental diets.

**Figure 7:**
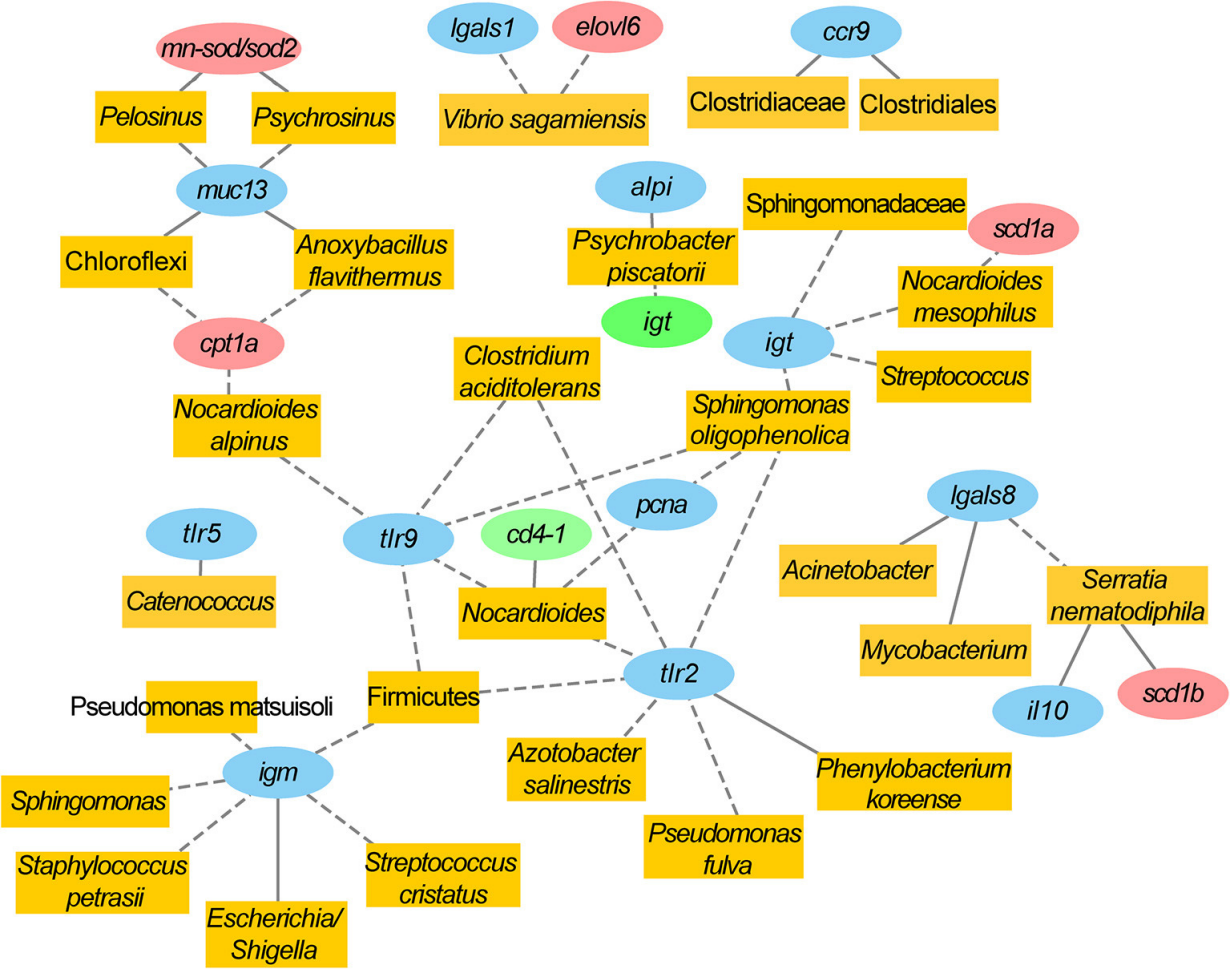
Correlation network showing significant positive (straight lines) and negative (dotted lines) correlations (Spearman, *P* < 0.01) between discriminant OTUs (yellow) and differentially expressed genes (DEG) in the anterior intestine (blue). Differentially expressed genes in the head kidney (green) and liver (red) with common interaction with anterior intestine gene—OTU correlations are also shown.

The DE genes at the intestinal level, together with those that came from the study of Fernandes et al. (2021) (liver, 12 DE; head kidney, 9 DE) were correlated with the normalized counts of the 135 discriminant OTUs (VIP ≥ 1). Gene expression patterns of selected markers of liver and head kidney with a high representation of markers of growth, lipid, and energy metabolism, antioxidant defense, and immune response are shown in Supplementary Tables 7, 8. In total, 4,590 correlations were performed, establishing 69 significant associations (*P* < 0.01) between 43 discriminant OTUs and 27 DE genes (Figure 6A). The 43 correlated bacteria represented ~8% in CTRL group, with the predominant OTUs being *Nocardioides alpinus* (2.24%) and *Sphingomonas oligophenolica* (1.11%). The percentage increased by up to 16.23% in fish fed with the non-PAP diet, with dominance of Clostridiales (8.6%), *Skermanella aerolata* (2.9%), Bacilli (1.62%), and *Serratia nematodiphila* (1%). In fish fed with the PAP diet, the associated OTUs represented 16.4%, with OTUs assigned to the *Escherichia/Shigella* (1.6%) and *Pelosinus* (1.12%) genera, and the *Psychrobacter piscatorii* (1.6%), *Haemophilus pittmaniae* (1.4%), and *Paracoccus yeei* (1.03%) species (Figure 6B). As a result of all this complex interplay, a remarkable number of DE genes at the intestinal level (12 out of 13) were involved in 38 significant correlations with 28 out of the 43 discriminant OTUs (VIP ≥ 1). Within these taxa, 10 OTUs disclosed a significant correlation with five DE genes of the liver and two of the head kidney (Figure 7). In a comparative manner, the correlation tests displayed eight discriminant OTUs that showed a significant correlation with seven hepatic DE genes (Supplementary Figure 5A), whereas seven discriminant OTUs were strongly associated with seven head kidney DE genes (Supplementary Figure 5B).

## Discussion

Fish production continues to be strongly dependent on FM (Ytrestøyl et al., 2015; Hua et al., 2019), but there is now evidence that the use of a combination of plant proteins, poultry meal, and insect proteins is able to support optimal growth in a wide range of farmed marine fish, such as gilthead sea bream (Basto et al., 2021; Reis et al., 2021). Moreover, there is now evidence that the reshaping of gut microbiota by the replacement of FM with poultry meal and microbial biomass would serve to exert an adaptive and counter-regulatory inflammatory action at the local intestine level (Solé-Jiménez et al., 2021). Certainly, a number of factors (e.g., age, sex, genetics, and environment) regulate the composition of the microbial population, which could eventually influence growth performance and health (Nayak, 2010; Cordero et al., 2015). However, diet-associated factors are perhaps one of the most important (Silva et al., 2011; Ghanbari et al., 2015). For instance, in zebrafish, a gluten-formulated diet displayed heightened abundances of Legionellales, Rhizobiaceae, and *Rhodobacter*, as compared with the control diet (Koo et al., 2017). Regarding aquaculture species, in yellowtail kingfish (*Seriola lalandi*), diet- and diet-associated bacteria shaped gut microbiota through development (Wilkes Walburn et al., 2019). Legume-based diets increased the abundance of lactic acid bacteria in Atlantic salmon, *Salmo salar* (Gajardo et al., 2017). Likewise, long-term feeding trials with plant-based diets displayed a shift in the resident intestinal microbiota of gilthead sea bream, driven by a dramatic increase in the genus *Photobacterium* that was partially reversed by dietary butyrate supplementation (Piazzon et al., 2017). However, interactions among microbial community, fish metabolism, fish performance, and health are still largely unknown, and this study serves to highlight the different reshaping of gut microbiota with the PAP and non-PAP diets. Additionally, several correlations between microbial populations and host gene expression were disclosed, with a more pronounced local intestinal action, and with important interactions at the systemic level.

At a closer look, the non-PAP diet decreased ACE and diversity Shannon indices, resulting in reduced richness and alpha diversity, in line with the study of Solé-Jiménez et al. (2021) using poultry meal and microbial biomass as FM replacements. The general thinking is that the occurrence of less diverse bacterial populations is a bad sign, because it is prone to increasing the chance of opportunistic pathogenic bacteria to proliferate and cause host damage (Sekirov et al., 2010; Apper et al., 2016). However, a deeper and experiment-specific understanding of the changes in gut microbiota is needed before reaching such conclusion for a given fish species and experimental condition. For instance, the gut microbiota of fish selected for growth became more stable but at the same time was functionally more plastic against changes in diet compositions (Piazzon et al., 2020). In this study, the microbial composition at the phylum level also remained relatively stable (Figure 3), showing the typical predominant phyla of gilthead sea bream within the expected range of variations for Proteobacteria (38–50% vs. 40–60%), Firmicutes (20–38% vs. 15–40%), and Bacteroidetes (3% vs. 2–5%), and only some deviations for Actinobacteria (11–18% vs. 15–25%) (Parma et al., 2016; Piazzon et al., 2019; Rimoldi et al., 2020). Since this occurred under brackish water conditions (18 ppt), this would be indicative that the gut microbiota of an euryhaline fish, such as gilthead sea bream, has the capacity to remain almost unaltered at the phylum level in the range of 18–37 ppt, which is in contrast with the dramatic changes observed with the sea water transfer in Atlantic salmon (Dehler et al., 2017). In this scenario, the PLS-DA disclosed 135 OTUs that drove (VIP ≥ 1) the separation among the groups (Figure 4). When this discriminant fraction of OTUs was considered more in-depth, the LEfSe analysis displayed a considerable number of taxa commonly related with opportunistic pathogenic bacteria in the gut of both the non-PAP- (Streptococcaceae, 3%; Flavobacteriaceae, 1%; *Sphingomonas*, 4.5%; *Staphylococcus*,2.5%) and PAP-fed fish (*Clostridium_*sensu_stricto, 5.5%) (Starliper, 2011; Sabry et al., 2016; Seghouani et al., 2017). However, assignments at high taxonomic levels, such as genus or families, do not necessarily reflect a pathogenic population, as these OTUs have been commonly detected in the microbial population of gilthead sea bream, and wide and diverse groups with several genus or species are implicated in diverse functions (Estruch et al., 2015; Parma et al., 2016; Nikouli et al., 2018; Piazzon et al., 2019).

Despite the increasing research on gut microbial composition, the understanding of how this population influences and is influenced by host gene expression remains relatively unexplored (Nichols and Davenport, 2021). Model organisms, such as zebrafish, *C. elegans, Drosophila melanogaster*, and mice are widely used to investigate these associations, with researchers having the control over the environment and complete control over diet, and importantly, the ability to study all tissues with advanced genomic tools (Borrelli et al., 2016; Wang et al., 2017). For aquaculture species, there is still a lack of information regarding the correlation between microbial populations and host gene expression, with some exceptions in black tiger shrimp (*Penaeus monodon*) (Uengwetwanit et al., 2020). Here, we take advantage of the genomic tools available for gilthead sea bream (Calduch-Giner et al., 2013; Pérez-Sánchez et al., 2019) to intersect the gene expression patterns of the intestine (from this study), liver, and head kidney (from Fernandes et al., 2021) with gut microbial community abundances (Figures 6, 7 and Supplementary Figure 5), putting together the paired samples from CTRL and fish fed with the PAP and non-PAP diets. It is worth noting that the high percentage of the OTUs (43 out of 135; ~32%) driving the separation among the dietary groups in the PLS-DA was significantly correlated with DE genes at the local and systemic levels. This fact supports the usefulness of the PLS-DA filter strategy (VIP ≥ 1) for the detection of transcriptome-associated taxa, as reported in other research animal models correlating metagenomics with host transcriptomics and metabolomics (Yan et al., 2020; Liu and Zhang, 2021; Zhao et al., 2021). Nonetheless, there is an important gap in information on the long-range action of the microbiota, mainly due to the assumption that microorganisms establish their niches in the intestine and they and their response do not spread, and that when an asystemic response exists, it is not always of sufficient clinical relevance (Chiu et al., 2017). However, both assumptions are questionable, because several studies stated the ability of resident microbiota to exert important systemic effects (Brenner et al., 2015; Ho et al., 2015; Grigg and Sonnenberg, 2017). Thus, in this fish study, the number of discriminant OTUs correlating with DE genes decreased from 28 in the intestine to 16 in the liver, and 9 in the head kidney. Among them, 10 out of 48 were involved in local and systemic correlations at the same time. According to this, the host and microbial interactions were more pronounced at the local (gut) level, but the systemic action cannot be underestimated, as further discussed below. Besides, our correlation analysis was based on a targeted gene approach with different tissue-specific PCR arrays, but it remains elusive if the use of a massive gene expression approach (RNA-seq) could modify the established trend for the network of gut microbiota and host transcriptomics.

The homoeostasis of the constantly renewing intestinal epithelium relies on integrated control of proliferation, differentiation, and apoptosis (Macara et al., 2014). Thus, the down-regulation of *pcna* in gilthead sea bream fed with FM-based diets supplemented with olive oil bioactive compounds has been related to a lower epithelial turnover in association with a better gut health condition (Gisbert et al., 2017). The *pcna* gene was also down-regulated in fish fed with plant-based diets, and the restoration of control values by butyrate supplementation was considered part of the mechanisms driven by this gut health factor in gilthead sea bream (Estensoro et al., 2016; Piazzon et al., 2017). Accordingly, in this study, the PAP-based feed formulation, and, only to a lower extent (non-significantly), the non-PAP diet (with a higher content of plant proteins), triggered the up-regulation of *pcna* in the AI of the fish in these two groups. This feature will be prone to promoting cell proliferation rather than cell differentiation with the replacement of FM with non-conventional dietary protein sources in the PAP and non-PAP diets. Of note, we found that the *pcna* gene expression was inversely correlated to *Nocardioides* (CTRL.003%; non-PAP and PAP <0.001%) and *Sphingomonas oligophenolica* (CTRL 2%; ~0.003% in the non-PAP and PAP fish), which suggests a role of these taxa in fish epithelial cell proliferation and regulation.

The integrity of the intestinal epithelium also relies on the maintenance of the mucus layer that is mainly composed of mucins, *O*-glycosylated glycoproteins that are present on the apex of all wet-surfaced epithelia, protecting epithelial cells from bacteria-, virus- or pH-derived damages and participating in cell signaling processes (Lang et al., 2007). Up to six mucins have been molecularly and transcriptionally characterized in gilthead sea bream, with *muc13* being extensively and constitutively expressed along the entire intestinal tract (Pérez-Sánchez et al., 2013). However, in this study, the expression of *muc13* was down-regulated in the non-PAP-fed fish, and to a lower extent (non-significantly), in the PAP fish. Besides, the correlation analysis highlighted a positive association of *muc13* with OTUs assigned to the phylum Chloroflexi (CTRL,0.58%; absent in PAP and non-PAP) and *Anoxybacillus flavithermus* (CTRL 0.01%; non-PAP and PAP <0.001%). Since Chloroflexi and the majority of species of the *Anoxybacillus* genus are described as anaerobic bacteria (Pikuta et al., 2000; Speirs et al., 2019), the up-regulation of *muc13* gene expression will tend to produce a thicker layer with less available oxygen that will favor intestinal colonization by these specific taxa. Intriguingly, we also evidenced a negative association of *muc13*mRNA transcripts with OTUs assigned to *Pelosinus* and *Psychrosinus* genera (CTRL, < 0.0001; NoPAP, 0.87%; PAP, 1.13%). To our knowledge, there is no described correlation between these taxa and host gene expression. However, species belonging to these taxa are capable of altering metal speciation, being well-known iron reducers (Ray et al., 2018). Besides, the higher bioavailability of iron in the intestinal lumen of mice has been related to the action of these bacteria on the reduction of intestinal oxidative DNA damage (Li et al., 2015; Eteshola et al., 2020). In our case, the up-regulated expression of the hepatic superoxide dismutase [Mn] (*mn-sod/sod2*) positively correlated with the higher abundance of *Pelosinus* and *Psychrosinus* genera in the AI of the non-PAP- and PAP-fed fish, pointing out a possible systemic cross-talk between fish antioxidant defense and gut microbial community.

As for *pcna*, the intestinal expression of *alpi* increased from the CTRL fish to the PAP fish, with intermediate expression values in the fish fed with the non-PAP diet. ALPI is found in high concentrations in the brush border of intestinal epithelial cells of both fish and mammals (Estensoro et al., 2016), regulating the rate of lipid absorption (Mahmood et al., 2003). Additionally, ALPI has a role in the disassembly of lipopolysaccharides (LPS) of Gram-negative bacteria (Poelstra et al., 1997), reducing inflammatory response (Millán, 2006; Rader, 2017). Thus, as in other animal models, a decline in ALPI activity is generally associated with malnutrition in fish (Bakke-McKellep et al., 2000; Ducasse-Cabanot et al., 2007), and perhaps to a less effective response for preventing bacterial invasion across the gut mucosal barrier. In this regard, we found that the up-regulated expression of *alpi* was closely related with a high representation of *Psychrobacter piscatorii* (CTRL and non-PAP <0.005%; PAP1.6%) in the intestinal microbiota of gilthead sea bream. Overall, this can be viewed as a positive feature, because some species of the *Psychrobacter* genus are tolerant to alkaline phosphatase (Denner et al., 2001), and their use as a probiotic reduced the proportion of harmful and pro-inflammatory species in the gastrointestinal tract of cultured orange-spotted grouper (*Epinephelus coloides*) (Yang et al., 2011). However, the species of this genus also activate the pro-inflammatory NF-kB pathway (Chow et al., 1999; Yu et al., 2016), and a recent study on gilthead sea bream (Solé-Jiménez et al., 2021) associated the lower abundance of *Psychrobacter* with the reshaping of gut microbiota to preserve an anti-inflammatory gut profile in fish fed with an FM replacement with a high content of poultry meal.

The expression of intestinal *igm* was markedly up-regulated in the AI of fish fed with the PAP diet. Earlier studies on this fish species highlighted the up-regulation of the intestinal *igm* in response to changes in the nutritional background, and bacteria or parasite challenges (Estensoro et al., 2012; Piazzon et al., 2016; Simó-Mirabet et al., 2017). Therefore, it is not surprising that this gene correlated with up to six different discriminant OTUs (i.e., Firmicutes, *Sphingomonas, Escherichia/Shigella, Pseudomonas matsuisoli, Staphylococcus petrasii*, and *Streptococcus cristatus*). Among these, only the OTUs assigned to the *Escherichia/Shigella* genus were found in a considerable proportion (1.6%) in the gut of fish fed with the PAP diet, establishing a direct correlation. This genus is defined as an activator of the inflammasome system (Liu et al., 2020), suggesting the driving role of this bacteria in the host immune response. In the head kidney, the expression trend for *igm* in the PAP fish was also up-regulated. However, the opposite was found for the *igt* gene expression, which showed a more down-regulated pattern in the head kidney than in the AI, which confirms and extends the idea of differential regulation of these two types of immunoglobulins in fish, gilthead sea bream in particular (Reyes-Cerpa et al., 2014; Piazzon et al., 2016). Besides, both the systemic and local *igt* expressions were negatively correlated with the abundance of intestinal *Psychrobacter piscatorii*, reinforcing the aforementioned relationship of this bacteria with gut health in gilthead sea bream.

Another pro-inflammatory marker, *il8*, was up-regulated in the AI of fish fed with the PAP diet, although in this case, we failed to establish any significant correlation with the intestinal microbial population. However, at the systemic level, the expression of the head kidney *il8* was negatively correlated with Bacilli. This link is not surprising, as bacteria from this class are known to have anti-inflammatory and anti-oxidant properties (Giri et al., 2019), being used as probiotics to improve growth and intestinal health in mammals (Rhayat et al., 2019; Zhou et al., 2020) and livestock fish (Mingmongkolchai and Panbangred, 2018), such as gilthead sea bream (Simó-Mirabet et al., 2017; Moroni et al., 2021). In this regard, it must be noted that the alleviation of the intestinal pro-inflammatory pattern of non-PAP fish was concomitant with a higher abundance of the OTUs assigned to Bacilli class (non-PAP 1.63%; CTRL and PAP <0.001%). In the head kidney, this was extended to the expression pattern of *tnf*α, which achieved the highest expression level in fish fed with the PAP diet and with intermediate values in the non-PAP fish. TNFα has a central role in inflammation (Bradley, 2008), and microbiota have been addressed to block (Yang et al., 2020) or activate its gene expression (Mendes et al., 2019). Hence, in the study, *tnf*α expression was positively correlated with the abundance of *Paracoccus yeei*, not present in the gut of CTRL fish but increasing by up to 0.3 and 1.03% in the non-PAP and PAP fish, respectively. This might serve to exert a counter-regulatory inflammatory response that is supported by the inferred metagenomics pathway analysis, where *P. yeei* is the only contributor in the inferred over-representation of the TNF signaling pathway. Thus, although bacteria-host gene expression interactions can be sometimes difficult to interpret, the combination of different analyses provided support to the results.

Another sign of an anti-inflammatory profile in the non-PAP fish was the intestinal up-regulation of *il10*, a key anti-inflammatory cytokine used in both fish and mammals as an important marker for health status of the host (Levast et al., 2015; Piazzon et al., 2015). Some increase in the expression level of *il10* was also seen in the head kidney, although not significant, confirming the stronger modulation of this cytokine at the local level, as reported in earlier gilthead sea bream studies (Pérez-Cordón et al., 2014). Moreover, regarding microbiota and gene expression associations, the intestinal expression of *il10* positively correlated with the abundant *Serratia nematodiphila* in non-PAP fish (1.02%), while being poorly represented in the microbial population (<0.001%) of CTRL and PAP fish. In any case, the role of the *Serratia* genus in inflammatory processes remains controversial, because some species act as pro-inflammatory agents through protease-activated receptors (Kida et al., 2007), whereas others are anti-inflammatory factors by means of enzymes with potent anti-oxidant power (Saeki et al., 1974; El-Abd and Ibrahim, 2020).

In a healthy gut, the recognition of microorganisms by PRRs has a primary role in the activation or repression of innate immunity (Fukata and Arditi, 2013), exerting diverse functions depending on the offending factor (Boltaña et al., 2011). In our experiment model, several PRRs, such as galectins and toll-like receptor genes, were monitored. Among them, it is worth noting that the pronounced down-regulation of intestinal *lgals8* in both PAP and non-PAP fish would contribute to the maintenance of the repressed immune system. This is in line with several pro-inflammatory molecules abundantly produced in mice by LGAL8-stimulated endothelial cells (Cattaneo et al., 2014). Otherwise, the intestinal expression of *lgals8* was significantly down-regulated in healthy gilthead sea bream juveniles fed with practical diets that are supplemented with sodium salt medium-chain fatty acids or *Bacillus*-based probiotic (Simó-Mirabet et al., 2017). Conversely, LGALs1 has a recognized role in the control of chronic inflammations, weakening cytokine synthesis and de-activating antigen presenting cells, causing an overall immune repression in mice (Seropian et al., 2018). Therefore, the up-regulated expression of intestinal *lgals1* in the non-PAP fish, but not in the PAP fish, reinforced the anti-inflammatory expression pattern of fish fed with the non-PAP diet. This was not the case for the *tlr* genes that showed an enhanced expression in the PAP and non-PAP fish (*tlr2*) or only in the NoPAP fish (*tlr9*). The functional significance of this finding remains unclear, but TLR signaling pathways play a key role in the regulation of the immune system, preventing autoimmune and inflammatory diseases in humans and rodents (Tang et al., 2012; Kawasaki and Kawai, 2014). Likewise, *tlr* genes are often up-regulated when fish deal with a bacterial infection (Reyes-Becerril et al., 2015), but there is now evidence that spirulina feed significantly enhanced the immune response of gibel carp (*Carassius auratus gibelio*) through the Tlr2 pathway (Cao et al., 2018). Since the non-PAP-based feed formulation also contained *Spirulina* as a microalgae meal, it is likely that the enhanced expression of *tlrs* in fish fed with the non-PAP diet was due, at least in part, to the use of this alternative dietary protein source. In any case, the number of OTUs (eight) associated with changes in the expression pattern of PPRs was relatively high. Besides, the correlated taxa belonged to different and diverse assignations (Firmicutes, *Clostridium, Nocardioides, Azotobacter, Pseudomonas*, and *Phenylobacterium*), showing the wide range of bacterial types that could be interacting with PRRs.

In a back-and-forth response, gut microbiota can exert a substantial influence on host lipid metabolism, and mechanistic links involving the microbial generation of SCFAs, microbial processing of bile acids, and bacterial-derived pro-inflammatory factors have been reported in mouse models (Schoeler and Caesar, 2019; Lamichhane et al., 2021). This highlights a gut microbiota-liver axis where SCFAs generated by the gut bacterial fermentation of dietary fiber fuel the SCD1 (Stearoyl CoA desaturase)-mediated lipogenesis in the liver of mice (Singh et al., 2015). Intriguing associations between host lipid metabolism and the composition of gut microbiota have also been reported in Atlantic salmon (Dvergedal et al., 2020) and rainbow trout (Yildirimer and Brown, 2018). However, in fish, these relationships remain in an infancy state, reinforcing the value of this study where, for the first time in a typical marine fish, a possible link between enzymes of lipid metabolism and gut microbiota was evidenced. Here, this can be exemplified by the lipogenic *scd1a* and *scd1b* enzymes, which becomes especially interesting in the case of *scd1a* given that a recent study revealed reliable epigenetic mechanisms (changes in DNA methylation rates of the *scd1a* promoter) by which the nutrition of parents can shape the s*cd1a* gene expression in the gilthead sea bream offspring (Perera et al., 2020). The extent to which this can be favored by changes in the gut microbiota remains unknown, but interestingly, the up-regulation of *scd1* genes in the fish fed with the non-PAP diet was associated with OTUs assigned to *Serratia nematodiphila* (NoPAP, 1.02%) and *Brevinema andersonii* (NoPAP 0.1%; CTRL and PAP <0.01%), giving these taxa a role as intestinal SCFA producers in humans (Parada Venegas et al., 2019) and enhancers of fatty acid metabolism in insects (Zhou et al., 2021). The case of *B. andersonii* merits further attention, as this species belongs to Spirochaetesphylum, which colonizes the gut gilthead sea bream microbiome to a large extent with advancing age (11% in 4-year-old individuals) (Piazzon et al., 2019). The physiological consequences of this finding merit further research, because studies on mice highlighted alterations of the hepatic lipid profile and fatty acid synthesis in response to gut colonization by aged microbiota (Albouery et al., 2020). Future studies should focus on enzymatic levels and activities to corroborate these results further.

Like hepatic SCD1, fatty acid elongase 6 (ELOVL6) is a rate-limiting enzyme of fatty acid synthesis and lipogenic activity, being triggered by the gut microbial production of SCFAs in mice (Kindt et al., 2018). This might also occur in our gilthead sea bream model, and the hepatic expression of *elovl6* was positively correlated with the non-pathogenic species *Staphylococcus carnosus* (non-PAP 0.03%; PAP <0.00001%), applied as a starter culture in industrialized processes (Janssens et al., 2012). Another type of association of lipid metabolism and gut microbiota was represented by the peroxisome proliferator-activated receptor α (*ppar*α). This transcription factor of lipid metabolism is a well-known lipolytic factor (Mottillo et al., 2012; Pawlak et al., 2015), and its hepatic down-regulated expression in the fish fed with the non-PAP diet negatively correlated with OTUs assigned to Moraxellaceae, *Haemophillus pittmaniae*, and *Celeribacter marinus*. Therefore, the trade-off of host lipogenic and lipolytic pathways becomes related to the changes in the gut microbial population of a farmed marine fish.

**Figure 8:**
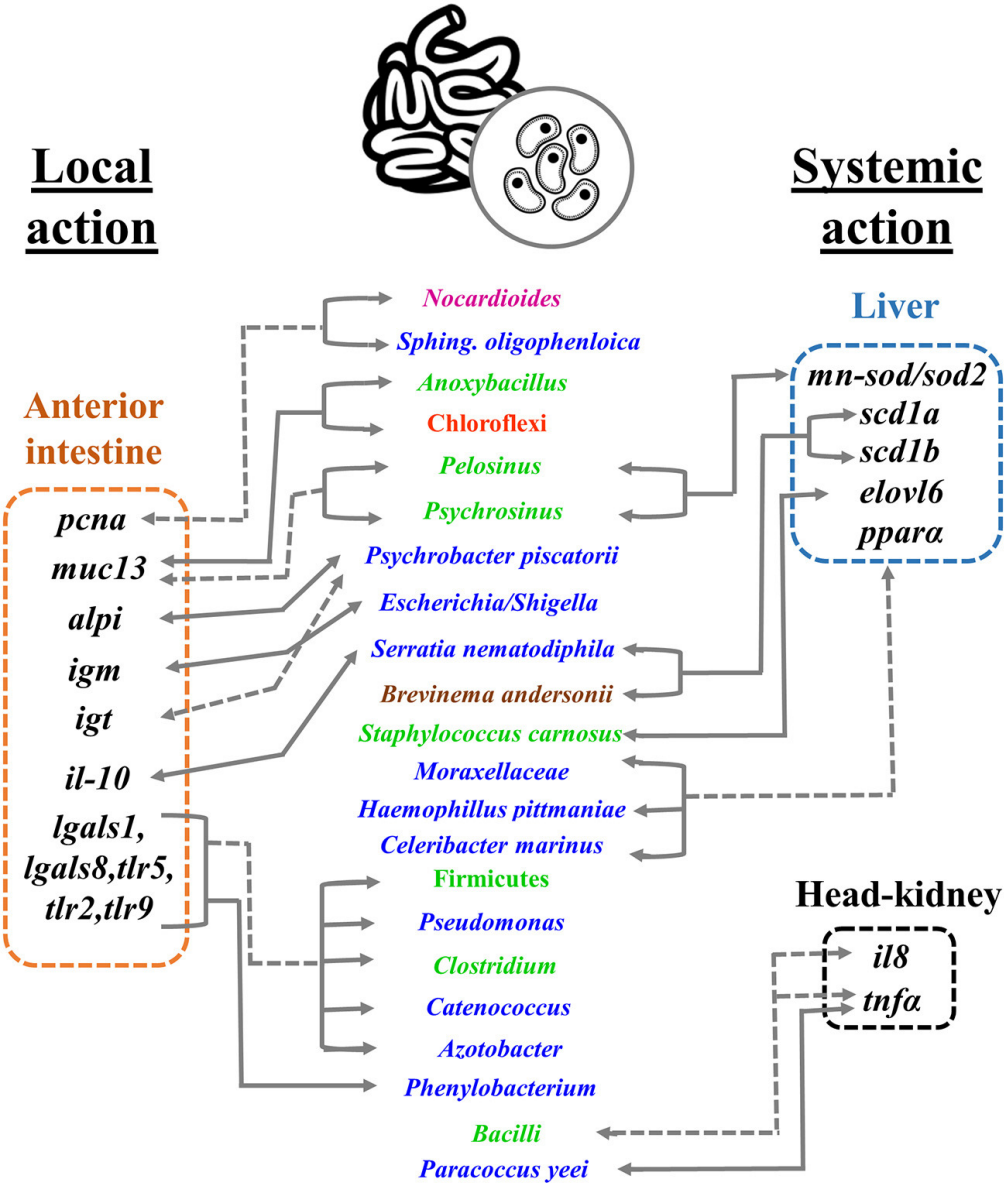
Schematic representation of the main significant correlations between gut microbial community abundances and host gene expression at the local (AI) and systemic levels (liver, head-kidney). Gene symbols are boxed in dashed rectangles referring to each tissue: anterior intestine (orange), liver (blue), and head-kidney (black). Taxa names are in the middle column, with colors representing the phyla they belong: Proteobacteria (blue), Firmicutes (green), Actinobacteria (pink), Chloroflexi (red), and Spirochaetes (brown). Gray lines between taxa and gene symbol represent a positive (straight) or negative (dashed) correlation between gene and OTU.

In summary, even in the absence of major changes in growth performance, a number of processes related to epithelial cell turnover, immune response, and lipid metabolism were affected by dietary intervention. Both pro- and anti-inflammatory responses were triggered by the non-PAP- and PAP-based feed formulations, although the net effect would be prone to a slight pro-inflammatory status that appears mostly attenuated in the fish fed with the non-PAP diet. Thus, the formulation of the non-PAP diet arises as an attractive formulation to be further studied for its use in gilthead sea bream aquaculture. Indeed, the fish fed with the non-PAP diet shared an enhanced lipogenic activity, which might suggest an enhanced microbial production of SCFAs. As summarized in Figure 8, remarkable correlations between changes in gut microbial populations and host gene expression were unveiled at the local and systemic levels. By correlating microbiome and host gene expression, we offer new insights into the physiological processes promoting both metabolic and gut homeostasis and, ultimately, the health of farmed fish. All this reinforces the action of the gut microbiome as a “second genome,” being involved in—and/or being influenced by—the regulation of the transcriptomic response in fish fed with new feed formulations based on increased circularity and resource utilization. Future studies should focus on the effect of improved non-PAP-based formulations on fish growth and health, with a special focus on disease resistance.

## Data Availability Statement

The datasets presented in this study can be found in the Sequence Read Archive (SRA) under Bioproject accession number PRJNA745265 (BioSample accession numbers: SAMN20157689-709).

## Ethics Statement

The animal study was reviewed and approved by Fernando Bernardo, Direçao Geral de Alimentaçao e Veterinaria, Lisboa, Portugal.

## Author Contributions

FN-C, GV, MP, AF, and JC-G: formal analysis. FN-C, GV, MP, and JP-S: writing—original manuscript. AS-B, LC, and JP-S: conceptualization. All authors contributed to the experimental investigation, writing—review and editing, read, and approved the final version of the manuscript.

## Funding

This study was supported by the EU H2020 GAIN Project (Aquaculture intensification in Europe, contract 773330). This study reflects only the views of the authors, and the European Union cannot be held responsible for any use that may be made of the information contained herein. Additional funding was obtained by the EU H2020 Research Innovation Program under the TNA Program (project AE150004) at the IATS-CSIC Research Infrastructure within the AQUAEXCEL^2020^ Project (652831), and the Spanish MICINN Project (Bream-AquaINTECH, RTI2018–094128-B-I00). MP was funded by a Ramón y Cajal Postdoctoral Research Fellowship (RYC2018-024049-I/AEI/10.13039/501100011033, co-funded by the European Social Fund (ESF) and ACOND/2020 Generalitat Valenciana).

## Conflict of Interest

GV, AF, and LC were employed by the company SPAROS (Olhăo, Portugal). The remaining authors declare that the research was conducted in the absence of any commercial or financial relationships that could be construed as a potential conflict of interest.

## Publisher's Note

All claims expressed in this article are solely those of the authors and do not necessarily represent those of their affiliated organizations, or those of the publisher, the editors and the reviewers. Any product that may be evaluated in this article, or claim that may be made by its manufacturer, is not guaranteed or endorsed by the publisher.
