# Supplementary material for: Cross-Talk Between Intestinal Microbiota and Host Gene Expression in Gilthead Sea Bream (Sparus aurata) Juveniles: Insights in Fish Feeds for Increased Circularity and Resource Utilization
Source: Front Physiol. 2021 Oct 5;12:748265. doi: 10.3389/fphys.2021.748265 (PMC8523787; doi:10.3389/fphys.2021.748265)
Supplement: Supplementary file 1 [file Table_1.DOCX]

**Supplementary Table 1**. Gilthead sea bream primers for qPCR amplification of intestinal genes.

| **Gene** | **Symbol** | | **GenBank** | | **Primer** | |
| --- | --- | --- | --- | --- | --- | --- |
|  |  | |  | |  |  |
| Proliferating cell nuclear antigen | *pcna* | KF857335 | | F: CGT ATC TGC CGT GAC CTG T | | |
|  |  |  |  | R: AGA ACT TGA CTC CGT CCT TGG | | |
|  |  |  | |  | |  |
| Transcription factor HES-1-B | *hes1-b* | KF857344 | | F: GCC TGC CGA TAT GAT GGA A | | |
|  |  |  |  | R: GGA GTT GTG TTC ATG CTT GC | | |
|  |  |  | |  | |  |
| Krueppel-like factor 4 | *klf4* | KF857346 | | F: ACA TCA CCG CAC GCA CAC | | |
|  |  |  |  | R: AAC CAC AGC CCT CCC AGT C | | |
|  |  |  | |  | |  |
| Claudin-12 | *cldn12* | KF861992 | | F: CTC TCA GGG CTA CAC ATC TAC CTA TGC | | |
|  |  |  |  | R: ACA TTC GTG AGC GGC TGG AG | | |
|  |  |  | |  | |  |
| Claudin-15 | *cldn15* | KF861993 | | F: CCG ATT GTG GAA GTA GTG GCT CTG GT | | |
|  |  |  |  | R: CAG CAT CAC CCA ACC GAC GAA CC | | |
|  |  |  | |  | |  |
| Cadherin-1 | *cdh1* | KF861995 | | F: TGC TCC ATA CAG CGT CAC CTT ACA | | |
|  |  |  |  | R: CTC GTT CAT CCT AGC CGT CCA GTT | | |
|  |  |  | |  | |  |
| Cadherin-17 | *cdh17* | KF861996 | | F: GAT GCC CGC AAC CCA GAG | | |
|  |  |  |  | R: CCG TTG ATT CAC TGC CGT AGA C | | |
|  |  |  | |  | |  |
| Tight junction protein ZO-1 | *tjp1* | KF861994 | | F: AAG CAG TAT TAC GGT GAC TCA | | |
|  |  |  |  | R: TGC ATC CCT GGC TTG TAG | | |
|  |  |  | |  | |  |
| Desmoplakin | *dsp* | KF861999 | | F: GCA GAA GGA GCA CGA GAC CATC | | |
|  |  |  |  | R: GGG TGT TCT TGT CGC AGG TGA A | | |
|  |  |  | |  | |  |
| Gap junction Cx32.2 protein | *cx32.2* | KF862000 | | F: CGA GGT GTT CTA TCT GCT CTG TA | | |
|  |  |  |  | R: CTT GTG GGT GCG AGT CCT | | |
|  |  |  | |  | |  |
| Coxsackievirus and adenovirus receptor homolog | *cxadr* | KF861998 | | F: CAT CAG AGG ACT ACG AGA GG | | |
|  |  |  |  | R: CAT CTT GGC AGC ATT TGG T | | |
|  |  |  | |  | |  |
| Intestinal-type alkaline phosphatase | *alpi* | KF857309 | | F: CCG CTA TGA GTT GGA CCG TGA T | | |
|  |  |  |  | R: GCT TTC TCC ACC ATC TCA GTA AGG G | | |
|  |  |  | |  | |  |
| Liver type fatty acid-binding protein | *fabp1* | KF857311 | | F: GTC CTC GTC AAC ACC TTC ACC AT | | |
|  |  |  |  | R: CGC CTT CAT CTT CTC GCC AGT | | |
|  |  |  | |  | |  |
| Intestinal fatty acid-binding protein | *fabp2* | KF857310 | | F: CGA GCA CAT TCC GCA CCA AAG | | |
|  |  |  |  | R: CCC ACG CAC CCG AGA CTT C | | |
|  |  |  | |  | |  |
| Mucin 2 | *muc2* | JQ277710 | | F: ACG CTT CAG CAA TCG CAC CAT | | |
|  |  |  |  | R: CCA CAA CCA CAC TCC TCC ACA T | | |
|  |  |  | |  | |  |
| Mucin 13 | *muc13* | JQ277713 | | F: TTC AAA CCC GTG TGG TCC AG | | |
|  |  |  |  | R: GCA CAA GCA GAC ATA GTT CGG ATA T | | |
|  |  |  | |  | |  |
| Intestinal mucin | *i-muc* | JQ277712 | | F: GTG TGA CCT CTT CCG TTA | | |
|  |  |  |  | R: GCA ATG ACA GCA ATG ACA | | |
|  |  |  | |  | |  |
| Tumor necrosis factor-alpha | *tnfα* | AJ413189 | | F: CAG GCG TCG TTC AGA GTC TC | | |
|  |  |  |  | R: CTG TGG CTG AGA GCT GTG AG | | |
|  |  |  | |  | |  |
| Interleukin-1 beta | *il1β* | AJ419178 | | F: GCG ACC TAC CTG CCA CCT ACA CC | | |
|  |  |  |  | R: TCG TCC ACC GCC TCC AGA TGC | | |
|  |  |  | |  | |  |
|  |  |  | |  | |  |
| Interleukin-6 | *il6* | EU244588 | | F: TCT TGA AGG TGG TGC TGG AAG TG | | |
|  |  |  |  | R: AAG GAC AAT CTG CTG GAA GTG AGG | | |
|  |  |  | |  | |  |
| Interleukin-7 | *il7* | JX976618 | | F: CTA TCT CTG TCC CTG TCC TGT GA | | |
|  |  |  |  | R: TGC GGA TGG TTG CCT TGT AAT | | |
|  |  |  | |  | |  |
| Interleukin-8 | *il8* | JX976619 | | F: CAG CAG AGT CTT CAT CGT CAC TAT TG | | |
|  |  |  |  | R: AGG CTC GCT TCA CTG ATG G | | |
|  |  |  | |  | |  |
| Interleukin-10 | *il10* | JX976621 | | F: AAC ATC CTG GGC TTC TAT CTG | | |
|  |  |  |  | R: GTG TCC TCC GTC TCA TCT G | | |
|  |  |  | |  | |  |
| Interleukin 12 subunit beta | *il12* | JX976624 | | F: ATT CCC TGT GTG GTG GCT GCT | | |
|  |  |  |  | R: GCT GGC ATC CTG GCA CTG AAT | | |
|  |  |  | |  | |  |
| Interleukin 15 | *il15* | JX976625 | | F: GAG ACC AGC GAG CGA AAG GCA TCC | | |
|  |  |  |  | R: GCC AGA ACA GGT TAC AGG TTG ACA GGA A | | |
|  |  |  | |  | |  |
| Interleukin 34 | *il34* | JX976629 | | F: TCT GTC TGC CTG CTG GTA G | | |
|  |  |  |  | R: ATG CTG GCT GGT GTC TGG | | |
|  |  |  | |  | |  |
| CD4-1 | *cd4-1* | AM489485 | | F: TCCTCCTCCTCGTCCTCGTT | | |
|  |  |  |  | R: GGTGTCTCATCTTCCGCTGTCT | | |
|  |  |  | |  | |  |
| CD8 beta | *cd8β* | KX231275 | | F: CCGAAATGTGGAAGACTGGAACTC | | |
|  |  |  |  | R: CTTTGGAGGTAAGGTTGGAGGGAT | | |
|  |  |  | |  | |  |
| C-C chemokine receptor type 3 | *ccr3* | KF857317 | | F: CTA CAT CAG CAT CAC CAT ACG CAT CCT | | |
|  |  |  |  | R: TGG CAC GGC ACT TCT CCT TCA | | |
|  |  |  | |  | |  |
| C-C chemokine receptor type 9 | *ccr9* | KF857318 | | F: TCC CTG AGT TAA TCT TCG CCC AAG TG | | |
|  |  |  |  | R: TGT TGT ATT CGT TGT TCC AGT AGA CCA GAG | | |
|  |  |  | |  | |  |
| C-C chemokine receptor type 11 | *ccr11* | KF857319 | | F: GCT ACG ATT ACA GTT ATG AA | | |
|  |  |  |  | R: TAG ATG ATT GGG AGG AAG | | |
|  |  |  | |  | |  |
| C-C chemokine CK8 / C-C motif chemokine 20 | *ck8/ cl20* | GU181393 | | F: CCG TCC TCA TCT GCT TCA TAC T | | |
|  |  |  |  | R: GCT CTG CCG TTG ATG GAA C | | |
|  |  |  | |  | |  |
| Macrophage colony-stimulating factor 1 receptor 1 | *csf1r1* | AM050293 | | F: TTG CGT GTG GTG AGG AAG GAA GGT | | |
|  |  |  |  | R: AGC AGG CAG GGC AGC AGG TA | | |
|  |  |  | |  | |  |
| Immunoglobulin M | *igm* | JQ811851 | | F: ACC TCA GCG TCC TTC AGT GTT TAT GAT GCC | | |
|  |  |  |  | R: CAG CGT CGT CGT CAA CAA GCC AAG C | | |
|  |  |  | |  | |  |
| Immunoglobulin T | *igt* | KX599201 | | F: AGA CGA TGC CAG TGA AGA GGA TGA GT | | |
|  |  |  |  | R: CGA AGG AGG AGG CTG TGG ACC A | | |
|  |  |  | |  | |  |
| Galectin-1 | *lgals1* | KF862003 | | F: GTG TGA GGA GGT CCG TGA TG | | |
|  |  |  |  | R: ACT GTA GAG CCG TCC GAT AGG | | |
|  |  |  | |  | |  |
| Galectin-8 | *lgals8* | KF862004 | | F: GGC GGT GAA CGG CGG TCA | | |
|  |  |  |  | R: GCT CCA GCT CCA GTC TGT GTT GAT AC | | |
|  |  |  | |  | |  |
| Toll-like receptor 2 | *tlr2* | KF857323 | | F: CAT CTG CGA CTC TCC TCT CTT CCT | | |
|  |  |  |  | R: ATT CAA CAA TGG AGC GGT GGA CTT | | |
|  |  |  | |  | |  |
| Toll-like receptor 5 | *tlr5* | KF857324 | | F: TCG CCA ATC TGA CGG ACC TGA G | | |
|  |  |  |  | R: CAG AAC GCC GAT GTG GTT GTA AGA C | | |
|  |  |  | |  | |  |
| Toll-like receptor 9 | *tlr9* | AY751797 | | F: GCC TTC CTT GTC TGC TCT TTC T | | |
|  |  |  |  | R: GCC GTA GAG GTG CTT CAG TAG | | |
|  |  |  | |  | |  |
| C-type lectin domain family 10 member A | *clec10a* | KF857329 | | F: CGA CTC TGG ACT CCC TCA | | |
|  |  |  |  | R: CGT TGT TGA TGG TGC GTT C | | |
|  |  |  | |  | |  |
| Macrophage mannose receptor 1 | *mrc1* | KF857326 | | F: CTT CCG ACC GTA CCT GTA CCT ACT CA | | |
|  |  |  |  | R: CGA TTC CAG CCT TCC GCA CAC TTA | | |
|  |  |  | |  | |  |
| Fucolectin | *fcl* | KF857331 | | F: CCA TAC TGC TGA ACA GAC CAA CC | | |
|  |  |  |  | R: TGA TGG AGG TGA CGA TGT AGG A | | |
