# Supplementary material for: Cross-Talk Between Intestinal Microbiota and Host Gene Expression in Gilthead Sea Bream (Sparus aurata) Juveniles: Insights in Fish Feeds for Increased Circularity and Resource Utilization
Source: Front Physiol. 2021 Oct 5;12:748265. doi: 10.3389/fphys.2021.748265 (PMC8523787; doi:10.3389/fphys.2021.748265)
Supplement: Supplementary file 2 [file Table_2.DOCX]

**Supplementary Table 2.** Data on growth performance of CTRL and fish fed NoPAP- and PAP-based feed formulations. Data are the mean ± SEM of quadruplicate tanks for the whole population and fish used in microbiota and gene expression analyses (Modified from Fernandes et al. 2021).

|  | **CTRL** | **NoPAP** | **PAP** | ***P*^1^** |
| --- | --- | --- | --- | --- |
| *Whole population* |  |  |  |  |
| Initial body weight (g) | 56.2±0.8 | 55.8±0.33 | 55.8±0.35 | 0.73 |
| Final body weight (g) | 136.7±3.27 | 134.6±6.35 | 128.4±3.88 | 0.070 |
| Voluntary feed intake (g DM/fish) | 1.63±0.06 | 1.70±0.11 | 1.76±0.07 | 0.082 |
| FCR^2^ | 1.40±0.02 (c) | 1.48±0.03 (b) | 1.62±0.02 (a) | **<0.0001** |
|  |  |  |  |  |
| *Microbiota/gene expression* |  |  |  |  |
| Final body weight (g) | 130.1±4.14 | 131.8±4.15 | 128.8±3.41 | 0.86 |

^1^P values result from one-way ANOVA. Different superscript letters in each row indicate significant differences among dietary treatments (Student Newman-Keuls *P* < 0.1, bold values).

^2^FCR: Feed conversion ratio = dry feed intake/wet weight gain.
