# Supplementary material for: Cross-Talk Between Intestinal Microbiota and Host Gene Expression in Gilthead Sea Bream (Sparus aurata) Juveniles: Insights in Fish Feeds for Increased Circularity and Resource Utilization
Source: Front Physiol. 2021 Oct 5;12:748265. doi: 10.3389/fphys.2021.748265 (PMC8523787; doi:10.3389/fphys.2021.748265)
Supplement: Supplementary file 3 [file Table_3.DOCX]

**Supplementary Table 3**. Table showing the detailed sequencing data obtained in this study, as well as the number of observed OTUs, the species richness estimator (Chao1), and coverage (calculated from the ratio of observed OTUs and Chao1) obtained for each sample.

| **Group** | **Samples** | **Raw reads** | **Preprocessed reads** | **Joined and assigned reads** | **Observed OTUs** | **Chao1** | **Coverage (%)** |
| --- | --- | --- | --- | --- | --- | --- | --- |
|  |  |  |  |  |  |  |  |
| CTRL | 1 | 220491 | 214979 | 157854 | 266 | 326.41 | 81.49 |
| CTRL | 2 | 200632 | 195616 | 112286 | 182 | 243.00 | 74.90 |
| CTRL | 3 | 214507 | 209144 | 141807 | 265 | 365.64 | 72.48 |
| CTRL | 4 | 221455 | 215919 | 156266 | 261 | 346.58 | 75.31 |
| CTRL | 5 | 199632 | 195639 | 136947 | 69 | 301.25 | 22.9 |
| NoPAP | 1 | 106553 | 103889 | 70301 | 91 | 91.00 | 100.00 |
| NoPAP | 2 | 171031 | 166755 | 103514 | 84 | 245.00 | 34.29 |
| NoPAP | 3 | 154504 | 150641 | 104658 | 162 | 246.00 | 65.85 |
| NoPAP | 4 | 206991 | 201816 | 90903 | 134 | 202.50 | 66.17 |
| NoPAP | 5 | 189485 | 184748 | 138470 | 152 | 352.20 | 43.16 |
| NoPAP | 6 | 142020 | 138470 | 98063 | 79 | 116.00 | 68.10 |
| NoPAP | 7 | 223183 | 217603 | 164133 | 101 | 188.35 | 53.62 |
| NoPAP | 8 | 128166 | 124962 | 81342 | 121 | 121.00 | 100.00 |
| NoPAP | 9 | 174107 | 169754 | 116032 | 114 | 220.40 | 51.72 |
| PAP | 1 | 198826 | 193855 | 135360 | 197 | 379.00 | 51.98 |
| PAP | 2 | 206840 | 201669 | 147779 | 83 | 185.55 | 44.73 |
| PAP | 3 | 207403 | 202218 | 138149 | 109 | 298.10 | 36.56 |
| PAP | 4 | 201979 | 196930 | 139195 | 104 | 156.11 | 66.62 |
| PAP | 5 | 176784 | 172364 | 124524 | 95 | 252.00 | 37.70 |
| PAP | 6 | 195720 | 190827 | 124361 | 129 | 247.19 | 52.19 |
| PAP | 7 | 222491 | 216929 | 150417 | 130 | 250.75 | 51.84 |
