# Supplementary material for: Cross-Talk Between Intestinal Microbiota and Host Gene Expression in Gilthead Sea Bream (Sparus aurata) Juveniles: Insights in Fish Feeds for Increased Circularity and Resource Utilization
Source: Front Physiol. 2021 Oct 5;12:748265. doi: 10.3389/fphys.2021.748265 (PMC8523787; doi:10.3389/fphys.2021.748265)
Supplement: Supplementary file 6 [file Table_6.DOCX]

|  | **CTRL** | **NoPAP** | **PAP** | ***P*^1^** |
| --- | --- | --- | --- | --- |
| *pcna* | 2.73 ± 0.18^b^ | 3.01 ± 0.28^ab^ | 3.67 ± 0.30^a^ | **0.043**** |
| *hes1-b* | 1.17 ± 0.24 | 0.82 ± 0.16 | 1.02 ± 0.11 | 0.500 |
| *klf4* | 1.76 ± 0.13 | 1.83 ± 0.15 | 1.80 ± 0.11 | 0.131 |
| *cldn12* | 0.63 ± 0.05 | 0.70 ± 0.07 | 0.70 ± 0.05 | 0.663 |
| *cldn15* | 20.5 ± 2.80 | 18.6 ± 1.88 | 20.4 ± 1.93 | 0.913 |
| *cdh1* | 10.8 ± 0.69 | 10.5 ± 0.98 | 12.3 ± 0.88 | 0.137 |
| *cdh17* | 36.7 ± 3.33 | 30.5 ± 2.17 | 36.6 ± 2.11 | 0.218 |
| *tjp1* | 0.48 ± 0.03 | 0.49 ± 0.05 | 0.52 ± 0.03 | 0.557 |
| *dsp* | 4.25 ± 0.29 | 3.83 ± 0.63 | 3.84 ± 0.26 | 0.734 |
| *cx32.2* | 82.6 ± 8.56 | 66.9 ± 8.67 | 67.6 ± 6.31 | 0.516 |
| *cxadr* | 3.60 ± 0.21 | 3.54 ± 0.34 | 3.68 ± 0.24 | 0.357 |
| *alpi* | 32.3 ± 4.05 | 37.4 ± 3.95 | 48.6 ± 7.18 | 0.055 |
| *fabp1* | 91.5 ± 7.85 | 93.3 ± 10.1 | 86.2 ± 7.24 | 0.339 |
| *fabp2* | 499 ± 87.9 | 412 ± 97.5 | 416 ± 101 | 0.393 |
| *muc2* | 27.0 ± 4.21 | 20.8 ± 2.16 | 26.3 ± 2.92 | 0.502 |
| *muc13* | 68.3 ± 3.68^a^ | 53.7 ± 3.23^b^ | 60.4 ± 4.98^ab^ | **0.017**** |
| *i-muc* | 0.04 ± 0.01 | 0.08 ± 0.04 | 0.05 ± 0.01 | 0.436 |
| *tnfα* | 0.09 ± 0.01 | 0.11 ± 0.01 | 0.13 ± 0.01 | 0.118 |
| *il1β* | 0.14 ± 0.02 | 0.15 ± 0.02 | 0.14 ± 0.02 | 0.857 |
| *il6* | 0.02 ± 0.00 | 0.04 ± 0.01 | 0.02 ± 0.00 | 0.111 |
| *il7* | 0.34 ± 0.02 | 0.33 ± 0.02 | 0.30 ± 0.02 | 0.403 |
| *il8* | 0.18 ± 0.02^b^ | 0.20 ± 0.02^b^ | 0.27 ± 0.03^a^ | **0.004**** |
| *il10* | 0.08 ± 0.01 | 0.12 ± 0.02 | 0.09 ± 0.01 | **0.078*** |
| *il12* | 0.44 ± 0.03 | 0.37 ± 0.03 | 0.42 ± 0.05 | 0.494 |
| *il15* | 0.31 ± 0.03 | 0.28 ± 0.02 | 0.30 ± 0.03 | 0.718 |
| *il34* | 1.46 ± 0.12 | 1.65 ± 0.10 | 1.66 ± 0.10 | 0.222 |
| *cd4-1* | 0.21 ± 0.03 | 0.22 ± 0.04 | 0.22 ± 0.01 | 0.861 |
| *cd8β* | 0.04 ± 0.01 | 0.04 ± 0.00 | 0.04 ± 0.00 | 0.704 |
| *ccr3* | 0.94 ± 0.11 | 1.20 ± 0.15 | 1.14 ± 0.06 | 0.300 |
| *ccr9* | 1.43 ± 0.18^b^ | 2.22 ± 0.28^a^ | 1.46 ± 0.11^b^ | **0.016**** |
| *ccr11* | 6.37 ± 0.57 | 4.55 ± 0.60 | 5.83 ± 0.82 | 0.189 |
| *ck8 / ccl20* | 3.46 ± 0.34 | 2.44 ± 0.54 | 2.43 ± 0.17 | 0.210 |
| *csf1r1* | 0.64 ± 0.08 | 0.92 ± 0.09 | 0.88 ± 0.08 | 0.125 |
| *igm* | 5.39 ± 1.05^b^ | 11.6 ± 1.86^b^ | 36.5 ± 6.35^a^ | **<0.001***** |
| *igt* | 0.09 ± 0.01 | 0.11 ± 0.02 | 0.09 ± 0.01 | **0.074*** |
| *lgals1* | 6.47 ± 0.48^b^ | 16.2 ± 2.71^a^ | 8.91 ± 0.68^ab^ | **0.049**** |
| *lgals8* | 3.30 ± 0.55^a^ | 1.65 ± 0.18^b^ | 1.60 ± 0.15^b^ | **<0.001***** |
| *tlr2* | 0.34 ± 0.04 | 0.48 ± 0.05 | 0.47 ± 0.04 | **0.074*** |
| *tlr5* | 0.05 ± 0.01^a^ | 0.03 ± 0.00^b^ | 0.04 ± 0.01^ab^ | **0.048**** |
| *tlr9* | 0.04 ± 0.01^b^ | 0.11 ± 0.02^a^ | 0.09 ± 0.01^ab^ | **0.011**** |
| *clec10a* | 0.04 ± 0.01 | 0.04 ± 0.01 | 0.04 ± 0.00 | 0.301 |
| *mrc1* | 1.02 ± 0.11 | 1.35 ± 0.18 | 1.24 ± 0.07 | 0.156 |
| *fcl* | 4.95 ± 1.75 | 4.09 ± 2.64 | 7.78 ± 3.05 | 0.793 |

**Supplementary Table 6**. Relative gene expression of intestine genes in juvenile fish fed experimental diets. Data are the mean ± SEM of 9 fish. All data values for each tissue were in reference to the expression level of *hes1-b* of CTRL fish with an arbitrary assigned value of 1.

^1^ *P* values result from one-way ANOVA. Different superscript letters in each row indicate significant differences among dietary treatments (Student Newman-Keuls *P* < 0.1, bold values). Asterisks represent statistically significant differences at *P* < 0.1 (*), *P* < 0.05 (**) and *P* < 0.001 (***).
