# Supplementary material for: Cross-Talk Between Intestinal Microbiota and Host Gene Expression in Gilthead Sea Bream (Sparus aurata) Juveniles: Insights in Fish Feeds for Increased Circularity and Resource Utilization
Source: Front Physiol. 2021 Oct 5;12:748265. doi: 10.3389/fphys.2021.748265 (PMC8523787; doi:10.3389/fphys.2021.748265)
Supplement: Supplementary file 7 [file Table_7.DOCX]

**Supplementary Table 7**. Relative gene expression of hepatic genes in juvenile fish fed experimental diets. Data are the mean ± SEM of 10-12 fish. All data values for each tissue were in reference to the expression level of *cyp7a1* of CTRL fish with an arbitrary assigned value of 1.

|  | **CTRL** | **NOPAP** | **PAP** | **P^1^** |
| --- | --- | --- | --- | --- |
| *ghr-i* | 2.24 ± 0.25 | 2.23 ± 0.20 | 1.92 ± 0.18 | 0.320 |
| *ghr-ii* | 0.97 ± 0.07 | 0.98 ± 0.11 | 0.81 ± 0.11 | 0.470 |
| *igf-i* | 7.82 ± 0.83^b^ | 7.47 ± 0.76 ^b^ | 5.80 ± 0.53^a^ | **0.040**** |
| *igf-ii* | 2.77 ± 0.43 | 2.64 ± 0.62 | 2.23 ± 0.40 | 0.836 |
| *igfbp-1a* | 0.05 ± 0.01 | 0.04 ± 0.00 | 0.04 ± 0.01 | 0.118 |
| *igfbp-1b* | 6.09 ± 1.59 | 5.02 ± 1.62 | 5.19 ± 1.11 | 0.881 |
| *igfbp-2a* | 1.52 ± 0.13 | 1.49 ± 0.11 | 1.36 ± 0.11 | 0.802 |
| *igfbp-2b* | 1.67 ± 0.17 | 2.03 ± 0.14 | 1.54 ± 0.11 | 0.300 |
| *igfbp-4* | 0.58 ± 0.05 | 0.64 ± 0.04 | 0.59 ± 0.04 | 0.417 |
| *elovl1* | 7.90 ± 0.49 | 7.82 ± 0.58 | 6.66 ± 0.34 | 0.252 |
| *elovl4* | 0.18 ± 0.02^b^ | 0.19 ± 0.02^b^ | 0.12 ± 0.01^a^ | **0.036**** |
| *elovl5* | 2.53 ± 0.59^b^ | 2.49 ± 0.66 ^b^ | 0.81 ± 0.14^a^ | **0.022**** |
| *elovl6* | 1.71 ± 0.28 | 2.44 ± 0.40 | 1.32 ± 0.19 | **0.076*** |
| *fads2* | 2.04 ± 0.34 | 2.42 ± 0.37 | 1.60 ± 0.17 | 0.417 |
| *scd1a* | 0.16 ± 0.02^ab^ | 0.27 ± 0.06^b^ | 0.12 ± 0.01^a^ | **0.044**** |
| *scd1b* | 0.32 ± 0.06^a^ | 0.59 ± 0.13^b^ | 0.32 ± 0.10^ab^ | **0.050*** |
| *hl* | 7.93 ± 0.62^b^ | 7.61 ± 0.65^b^ | 5.25 ± 0.44^a^ | **0.007**** |
| *atgl* | 0.47 ± 0.11 | 0.42 ± 0.09 | 0.60 ± 0.20 | 0.688 |
| *lpl* | 8.09 ± 1.26 | 7.49 ± 0.85 | 7.58 ± 0.70 | 0.823 |
| *pla2g6* | 0.11 ± 0.01 | 0.13 ± 0.01 | 0.11 ± 0.01 | 0.113 |
| *cyp7a1* | 1.12 ± 0.18 | 1.33 ± 0.18 | 1.45 ± 0.22 | 0.653 |
| *pparα* | 2.22 ± 0.18^b^ | 1.46 ± 0.09^a^ | 1.61 ± 0.17^ab^ | **0.005**** |
| *pparβ* | 0.69 ± 0.09 | 0.80 ± 0.06 | 0.76 ± 0.10 | 0.694 |
| *pparγ* | 0.29 ± 0.02 | 0.32 ± 0.02 | 0.27 ± 0.02 | 0.156 |
| *cpt1a* | 0.71 ± 0.05^b^ | 0.59 ± 0.04^ab^ | 0.53 ± 0.05^a^ | **0.050*** |
| *h-fabp* | 45.2 ± 4.15 | 51.7 ± 3.54 | 43.1 ± 1.87 | 0.179 |
| *cs* | 0.51 ± 0.03^b^ | 0.48 ± 0.03^ab^ | 0.41 ± 0.03^a^ | **0.015**** |
| *sirt1* | 0.06 ± 0.01 | 0.06 ± 0.01 | 0.06 ± 0.00 | 0.711 |
| *sirt2* | 0.16 ± 0.01 | 0.15 ± 0.01 | 0.14 ± 0.01 | 0.489 |
| *ucp1* | 9.27 ± 0.94 | 11.0 ± 0.92 | 8.35 ± 0.70 | 0.123 |
| *gpx1* | 1.29 ± 0.07 | 1.15 ± 0.06 | 1.19 ± 0.11 | 0.674 |
| *gpx4* | 9.74 ± 1.14 | 11.4 ± 1.00 | 8.48 ± 0.82 | 0.123 |
| *prdx3* | 0.75 ± 0.06 | 0.87 ± 0.06 | 0.76 ± 0.07 | 0.309 |
| *prdx5* | 0.73 ± 0.06 | 0.69 ± 0.04 | 0.64 ± 0.06 | 0.652 |
| *cu-zn-sod/ sod1* | 3.53 ± 0.23 | 3.73 ± 0.18 | 3.36 ± 0.19 | 0.516 |
| *mn-sod / sod2* | 0.83 ± 0.06 | 1.13 ± 0.05 | 0.95 ± 0.09 | **0.050*** |
| *grp-170* | 1.00 ± 0.12 | 1.03 ± 0.09 | 0.80 ± 0.09 | 0.509 |
| *grp-94* | 3.51 ± 0.50 | 4.65 ± 0.39 | 3.72 ± 0.42 | 0.442 |
| *grp-75* | 0.47 ± 0.04 | 0.45 ± 0.03 | 0.43 ± 0.04 | 0.893 |
| *ctsb* | 1.75 ± 0.17 | 1.55 ± 0.09 | 1.38 ± 0.06 | **0.099*** |
| *ctsd* | 1.07 ± 0.08 | 1.21 ± 0.12 | 1.03 ± 0.09 | 0.344 |
| *ctsl* | 5.44 ± 0.56 | 6.14 ± 0.33 | 5.35 ± 0.49 | 0.618 |

^1^*P* values result from one-way ANOVA. Different superscript letters in each row indicate significant differences among dietary treatments (Student Newman-Keuls *P* < 0.1). Asterisks represent statistically significant differences at *P* < 0.1 (*) and *P* < 0.05 (**).
