# Supplementary material for: Cross-Talk Between Intestinal Microbiota and Host Gene Expression in Gilthead Sea Bream (Sparus aurata) Juveniles: Insights in Fish Feeds for Increased Circularity and Resource Utilization
Source: Front Physiol. 2021 Oct 5;12:748265. doi: 10.3389/fphys.2021.748265 (PMC8523787; doi:10.3389/fphys.2021.748265)
Supplement: Supplementary file 8 [file Table_8.DOCX]

**Supplementary Table 8**. Relative gene expression of head kidney in juvenile fish fed experimental diets. Data are the mean ± SEM of 10-12 fish. All data values for each tissue were in reference to the expression level of *il10* of CTRL fish with an arbitrary assigned value of 1.

|  | **CTRL** | **NOPAP** | **PAP** | | **P^1^** |
| --- | --- | --- | --- | --- | --- |
| *il1β* | 0.38 ± 0.08^a^ | 0.39 ± 0.04^a^ | 0.77 ± 0.09^b^ | | **0.005**** |
| *il6* | 0.07 ± 0.01 | 0.08 ± 0.02 | 0.09 ± 0.02 | | 0.170 |
| *il7* | 1.01 ± 0.11 | 1.02 ± 0.08 | 1.18 ± 0.08 | | 0.460 |
| *il8* | 0.09 ± 0.01^a^ | 0.22 ± 0.03^b^ | 0.25 ± 0.05^b^ | | **<0.001***** |
| *il10* | 1.01 ± 0.04 | 1.22 ± 0.10 | 1.09 ± 0.08 | | 0.233 |
| *il12* | 0.09 ± 0.01 | 0.09 ± 0.01 | 0.10 ± 0.01 | | 0.622 |
| *il15* | 0.34 ± 0.04 | 0.34 ± 0.02 | 0.36 ± 0.04 | | 0.867 |
| *il34* | 3.49 ± 0.31 | 3.31 ± 0.26 | 3.57 ± 0.16 | | 0.401 |
| *tnfα* | 0.24 ± 0.02 | 0.28 ± 0.02 | 0.34 ± 0.04 | | **0.030**** |
| *ccr3* | 7.02 ± 0.62^a^ | 8.26 ± 0.62^ab^ | 9.21 ± 0.39^b^ | | **0.044**** |
| *ck8/ccl20* | 1.57 ± 0.15^a^ | 1.96 ± 0.19^ab^ | 2.39 ± 0.33^b^ | | **0.027**** |
| *igm* | 264.3 ± 32.5 | 295.2 ± 28 | 314.2 ± 28 | | 0.175 |
| *igt-m* | 3.47 ± 0.58 | 3.44 ± 0.39 | 1.92 ± 0.32 | | **0.048**** |
| *a2m* | 0.11 ± 0.01 | 0.13 ± 0.03 | 0.15 ± 0.03 | | 0.300 |
| *b2m* | 143.8 ± 6.8 | 159.6 ± 11.8 | 136.6 ± 14.7 | 0.359 | |
| *c3* | 0.003 ± 0.001 | 0.004 ± 0.001 | 0.003 ± 0.001 | | 0.450 |
| *casp3* | 0.96 ± 0.05 | 1.18 ± 0.06 | 1.10 ± 0.08 | | 0.242 |
| *cd3ζ* | 3.33 ± 0.20^a^ | 3.92 ± 0.28^ab^ | 4.63 ± 0.41^b^ | | **0.049**** |
| *cd4-1* | 1.08 ± 0.09^a^ | 1.37 ± 0.10 ^ab^ | 1.73 ± 0.15^b^ | | **0.008**** |
| *cd8α* | 1.58 ± 0.09^b^ | 1.93 ± 0.23^ab^ | 2.27 ± 0.23^a^ | | **0.024**** |
| *cd8β* | 0.43 ± 0.04 | 0.54 ± 0.06 | 0.56 ± 0.06 | | 0.322 |
| *zap70* | 2.33 ± 0.20 | 2.84 ± 0.26 | 2.85 ± 0.30 | | 0.495 |
| *csf1r1* | 3.04 ± 0.25 | 3.42 ± 0.28 | 3.20 ± 0.21 | | 0.452 |
| *mrc1* | 13.8 ± 1.27 | 16.4 ± 2.19 | 15.0 ± 1.02 | | 0.725 |
| *tlr2* | 4.90 ± 0.37 | 6.15 ± 0.42 | 5.86 ± 0.42 | | 0.172 |
| *tlr5* | 1.17 ± 0.08 | 1.06 ± 0.06 | 1.07 ± 0.07 | | 0.473 |
| *tlr9* | 1.86 ± 0.19 | 2.18 ± 0.23 | 2.45 ± 0.15 | | 0.266 |
| *clec10a* | 2.48 ± 0.56 | 2.49 ± 0.55 | 2.93 ± 0.72 | | 0.950 |
| *fcl* | 0.06 ± 0.01 | 0.10 ± 0.02 | 0.06 ± 0.01 | | 0.286 |

^1^*P* values result from one-way ANOVA. Different superscript letters in each row indicate significant differences among dietary treatments (Student Newman-Keuls *P* < 0.1, bold values). Asterisks represent statistically significant differences at *P* < 0.05 (**) and *P* < 0.001 (***).
