# Supplementary material for: Cross-Talk Between Intestinal Microbiota and Host Gene Expression in Gilthead Sea Bream (Sparus aurata) Juveniles: Insights in Fish Feeds for Increased Circularity and Resource Utilization
Source: Front Physiol. 2021 Oct 5;12:748265. doi: 10.3389/fphys.2021.748265 (PMC8523787; doi:10.3389/fphys.2021.748265)

Supplementary Figure 1. Rarefaction curves obtained from the sequencing data of the 21 samples included in this study.

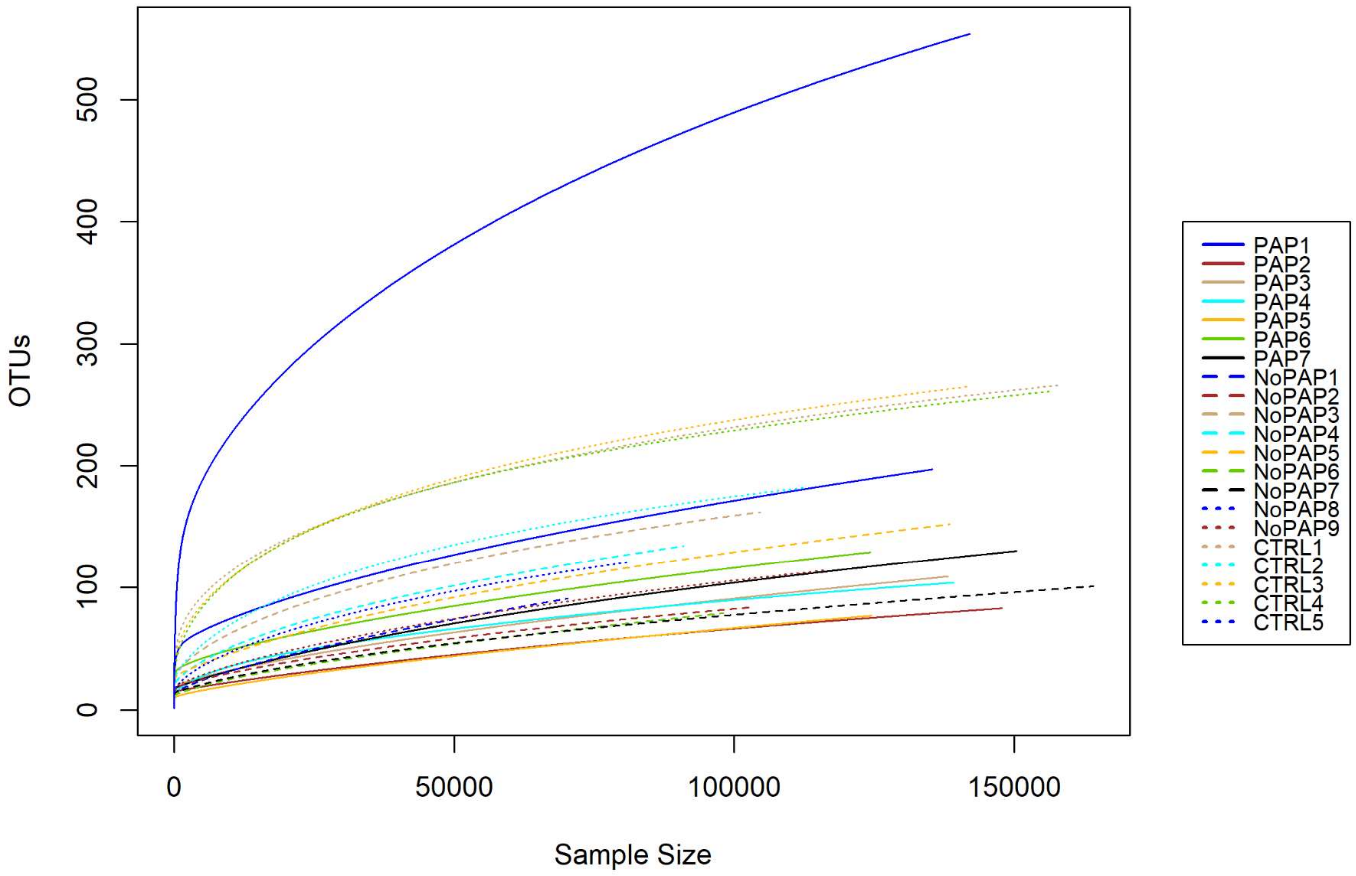

Supplement: Supplementary file 9 [file Image_1.pdf]
