# Supplementary material for: Cross-Talk Between Intestinal Microbiota and Host Gene Expression in Gilthead Sea Bream (Sparus aurata) Juveniles: Insights in Fish Feeds for Increased Circularity and Resource Utilization
Source: Front Physiol. 2021 Oct 5;12:748265. doi: 10.3389/fphys.2021.748265 (PMC8523787; doi:10.3389/fphys.2021.748265)

Supplementary Figure 4: Linear discriminant analysis effect size (LEfSe) performed with the discriminant OTUs (VIP ≥ 1) from the PLS-DA analysis. In (A) the OTUs most likely to drive differences in CTRL (black), NoPAP (red) and PAP (green) groups are represented in Log_{10} LDA score. These results are represented in a cladogram in (B) with the OTU classification at the level of phylum, class, order, family, genus and species shown from the inside to the outside.

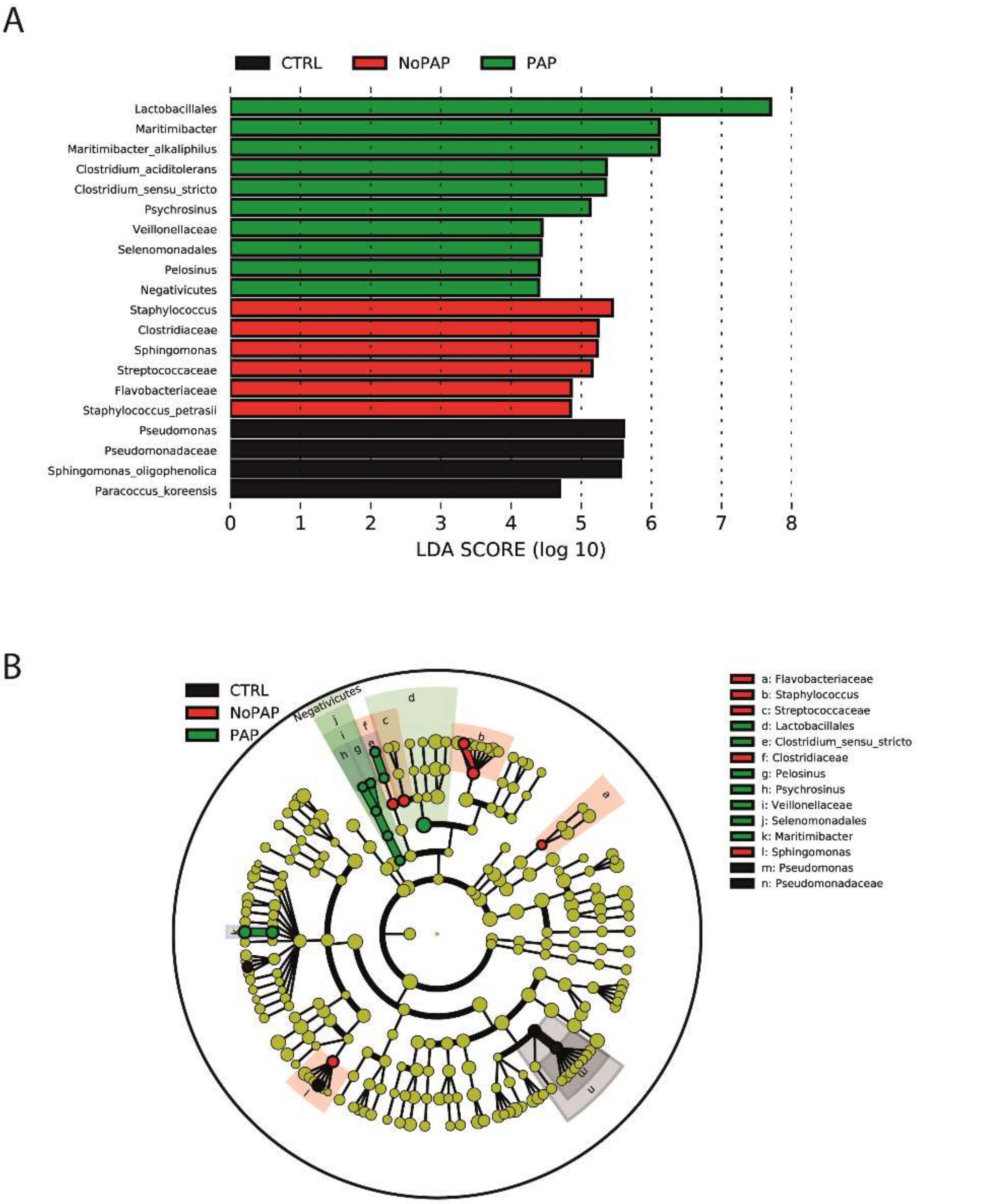

Supplement: Supplementary file 12 [file Image_4.pdf]
