# Supplementary material for: Cross-Talk Between Intestinal Microbiota and Host Gene Expression in Gilthead Sea Bream (Sparus aurata) Juveniles: Insights in Fish Feeds for Increased Circularity and Resource Utilization
Source: Front Physiol. 2021 Oct 5;12:748265. doi: 10.3389/fphys.2021.748265 (PMC8523787; doi:10.3389/fphys.2021.748265)

Supplementary Figure 5: Correlation network showing significant positive (straight lines) and negative (dotted lines) significant correlations (Spearman, P < 0.01) between discriminant OTUs (yellow) and differentially expressed genes (DEG) in (A) head kidney (green) and (B) liver (red).

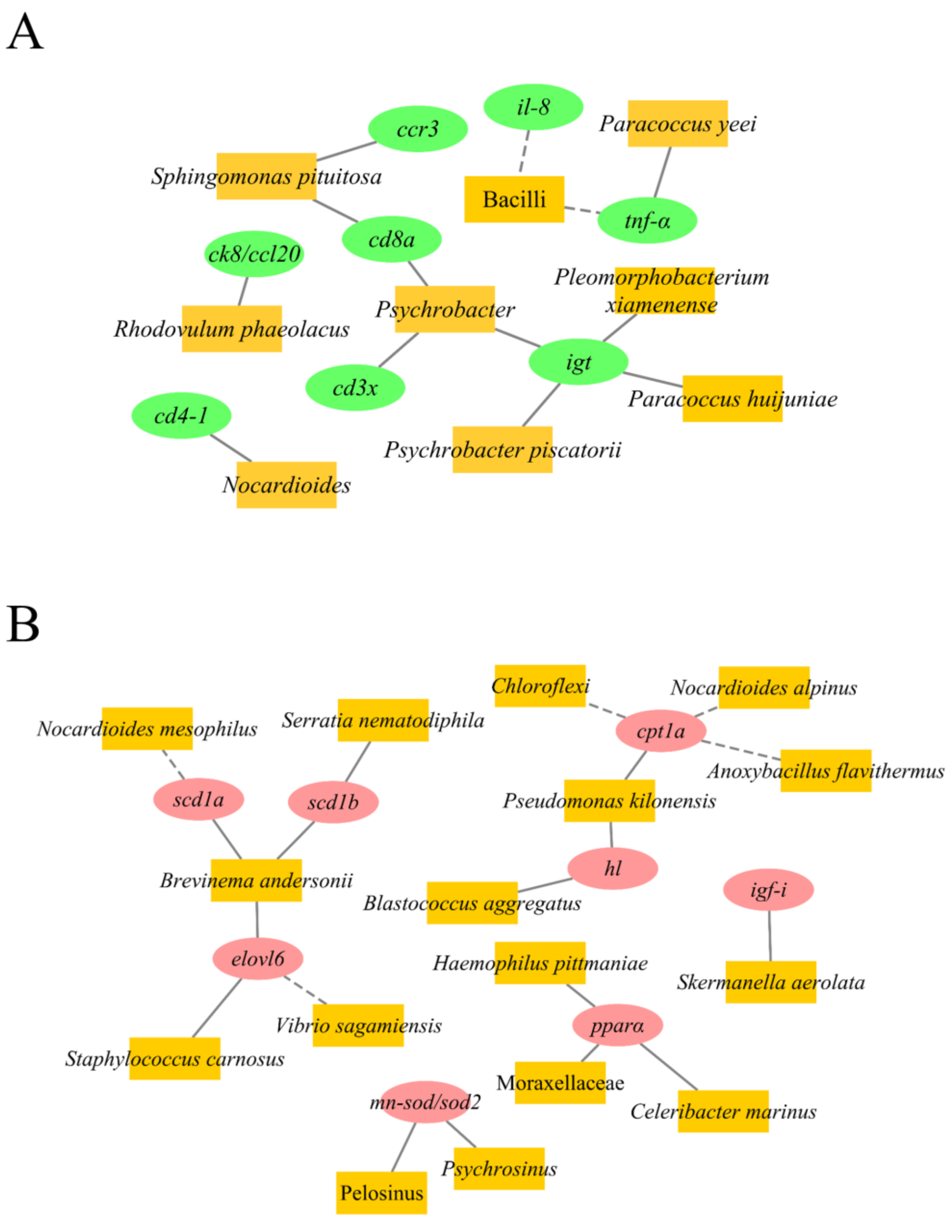

Supplement: Supplementary file 13 [file Image_5.pdf]
